# FAM20B-catalyzed glycosaminoglycans control murine tooth number by restricting FGFR2b signaling

**DOI:** 10.1186/s12915-020-00813-4

**Published:** 2020-07-14

**Authors:** Jingyi Wu, Ye Tian, Lu Han, Chao Liu, Tianyu Sun, Ling Li, Yanlei Yu, Bikash Lamichhane, Rena N. D’Souza, Sarah E. Millar, Robb Krumlauf, David M. Ornitz, Jian Q. Feng, Ophir Klein, Hu Zhao, Fuming Zhang, Robert J. Linhardt, Xiaofang Wang

**Affiliations:** 1 Southern Medical University Hospital of Stomatology, Guangzhou, 510280 Guangdong China; 2 Department of Biomedical Sciences, Texas A&M University College of Dentistry, 3302 Gaston Ave, Dallas, TX 75246 USA; 3 West China Hospital of Stomatology, Sichuan University, Chengdu, 610000 Sichuan China; 4 Department of Oral Pathology, College of Stomatology, Dalian Medical University, Dalian, 116044 Liaoning China; 5 Department of Developmental Biology, Washington University School of Medicine, St. Louis, MO 63110 USA; 6 Department of Chemistry and Chemical Biology, Rensselaer Polytechnic Institute, Troy, NY 12180 USA; 7 School of Dentistry, University of Utah, Salt Lake City, UT 84108 USA; 8 Department of Dermatology, Perelman School of Medicine, University of Pennsylvania, Philadelphia, PA 19104 USA; 9 Stowers Institute for Medical Research, Kansas City, MO 64110 USA; 10 Department of Anatomy and Cell Biology, Kansas University Medical Center, Kansas City, KS 66160 USA; 11 Department of Orofacial Sciences and Program in Craniofacial Biology, University of California, San Francisco, San Francisco, CA 94143 USA; 12 Institute for Human Genetics, University of California, San Francisco, San Francisco, CA 94143 USA

**Keywords:** Glycosaminoglycan, Proteoglycan, Fam20B, Kinase, Supernumerary teeth, Tooth renewal, Tooth replacement, Extracellular matrix, Stem cell, Sox2

## Abstract

**Background:**

The formation of supernumerary teeth is an excellent model for studying the molecular mechanisms that control stem/progenitor cell homeostasis needed to generate a renewable source of replacement cells and tissues. Although multiple growth factors and transcriptional factors have been associated with supernumerary tooth formation, the regulatory inputs of extracellular matrix in this regenerative process remains poorly understood.

**Results:**

In this study, we present evidence that disrupting glycosaminoglycans (GAGs) in the dental epithelium of mice by inactivating FAM20B, a xylose kinase essential for GAG assembly, leads to supernumerary tooth formation in a pattern reminiscent of replacement teeth. The dental epithelial GAGs confine murine tooth number by restricting the homeostasis of Sox2(+) dental epithelial stem/progenitor cells in a non-autonomous manner. FAM20B-catalyzed GAGs regulate the cell fate of dental lamina by restricting FGFR2b signaling at the initial stage of tooth development to maintain a subtle balance between the renewal and differentiation of Sox2(+) cells. At the later cap stage, WNT signaling functions as a relay cue to facilitate the supernumerary tooth formation.

**Conclusions:**

The novel mechanism we have characterized through which GAGs control the tooth number in mice may also be more broadly relevant for potentiating signaling interactions in other tissues during development and tissue homeostasis.

## Background

The ability to control stem/progenitor cell homeostasis is crucial for generating renewable source of replacement cells and tissues in regenerative medicine. A prerequisite for manipulating the renewal of stem cells is to understand the molecular mechanisms underlying the development of specific cell lineages and fates. The developing tooth organ is an excellent model system for studying the molecular mechanisms and signaling pathways that regulate organogenesis. The hierarchical interactions between the dental epithelium and underlying dental mesenchyme represent a common paradigm in the development of ectodermal placodes deployed in diverse types of epithelium organogenesis, such as salivary glands, *lungs*, *kidneys*, mammary glands, hair follicles, and limb buds [1]. Conserved signaling pathways, including those mediated by WNTs, bone morphogenetic proteins (BMPs), fibroblast growth factors (FGFs), and sonic hedgehog (SHH), are iteratively used in the cell-cell and cell-matrix communications during tooth development [2]. The secreted morphogens of these cascades interact with extracellular components, such as proteoglycans, to potentiate signal transduction. Proper cross-talk and a fine balance within these signaling pathways are critical for modulating the progressive temporal processes of tooth development, including tooth initiation, morphogenesis, and renewal. While proteoglycans have been identified in murine teeth at various embryonic stages [3–7], their precise inputs into tooth development remain poorly understood.

Proteoglycans consist of a core protein and one or multiple covalently attached glycosaminoglycan (GAG) chains. Based on the disaccharide structures, the polysaccharide GAGs can be classified into heparan sulfate (HS)/heparin, chondroitin sulfate (CS), dermatan sulfate (DS), keratan sulfate (KS), and hyaluronan (HA). Various sulfotransferases give rise to many sulfation patterns and modify the saccharide backbone of sulfated GAGs. The sulfated GAGs are among the most highly negatively charged biopolymers in nature, and variation in the sequence and length of the chains gives rise to enormous polydispersity. This rich structural diversity enables GAGs to interact with various proteins, including components of signaling cascades such as FGFs, WNTs, BMPs, and HHs that are involved in stem/progenitor cell homeostasis [8].

Family with sequence similarity member 20-B (FAM20B) is a kinase that specifically phosphorylates the xylose in the common linkage region of GAGs. The xylose phosphorylation is essential for the linkage region assembly and subsequent GAG elongation [9]. Inactivating FAM20B kinase leads to truncated polysaccharide chains that cannot be further elongated due to impaired function of galactosyl transferase II [10]. This fundamental property of FAM20B provides an useful tool for investigating the molecular functions of GAGs in organogenesis. Given that constitutive inactivation of *Fam20B* results in embryonic death at E13.5 [11], we generated a *Fam20B*-floxed allele in mice to facilitate conditional knockout studies. We found that inactivating *Fam20B* in the dental epithelium led to supernumerary incisors that were formed in a manner similar to replacement tooth formation [12], uncovering a previously unknown function of GAGs in the control of tooth number. Our results demonstrate that the FAM20B-catalyzed GAGs control the tooth number in mice by modulating the commitment of dental epithelial stem/progenitor cells through a mechanism involving the restriction of FGFR2b signaling at the initial stage of tooth development. Our findings provide novel insights into the molecular mechanism regulating tooth number and renewal in mice that may shed light on other GAG-mediated signaling events during organogenesis.

## Results

### GAG deficiency in the dental epithelium leads to supernumerary incisors in mice

It has been long known that proteoglycans are important molecules regulating signaling pathways during organogenesis. Decades ago, Thesleff et al. reported the expression of proteoglycans in developing murine teeth [13], and subsequent studies have identified multiple proteoglycans in both the dental epithelium and dental mesenchyme at various embryonic stages [6, 7, 14–16]. However, dissecting their mechanistic roles in tooth development has been challenging, because mice lacking individual proteoglycans or a particular type of GAGs did not show overt tooth phenotypes [17]. To explore this issue, we generated *Fam20B*-floxed mice to disrupt multiple GAGs in embryonic teeth in a tissue-specific manner.

**Fig. 1:**
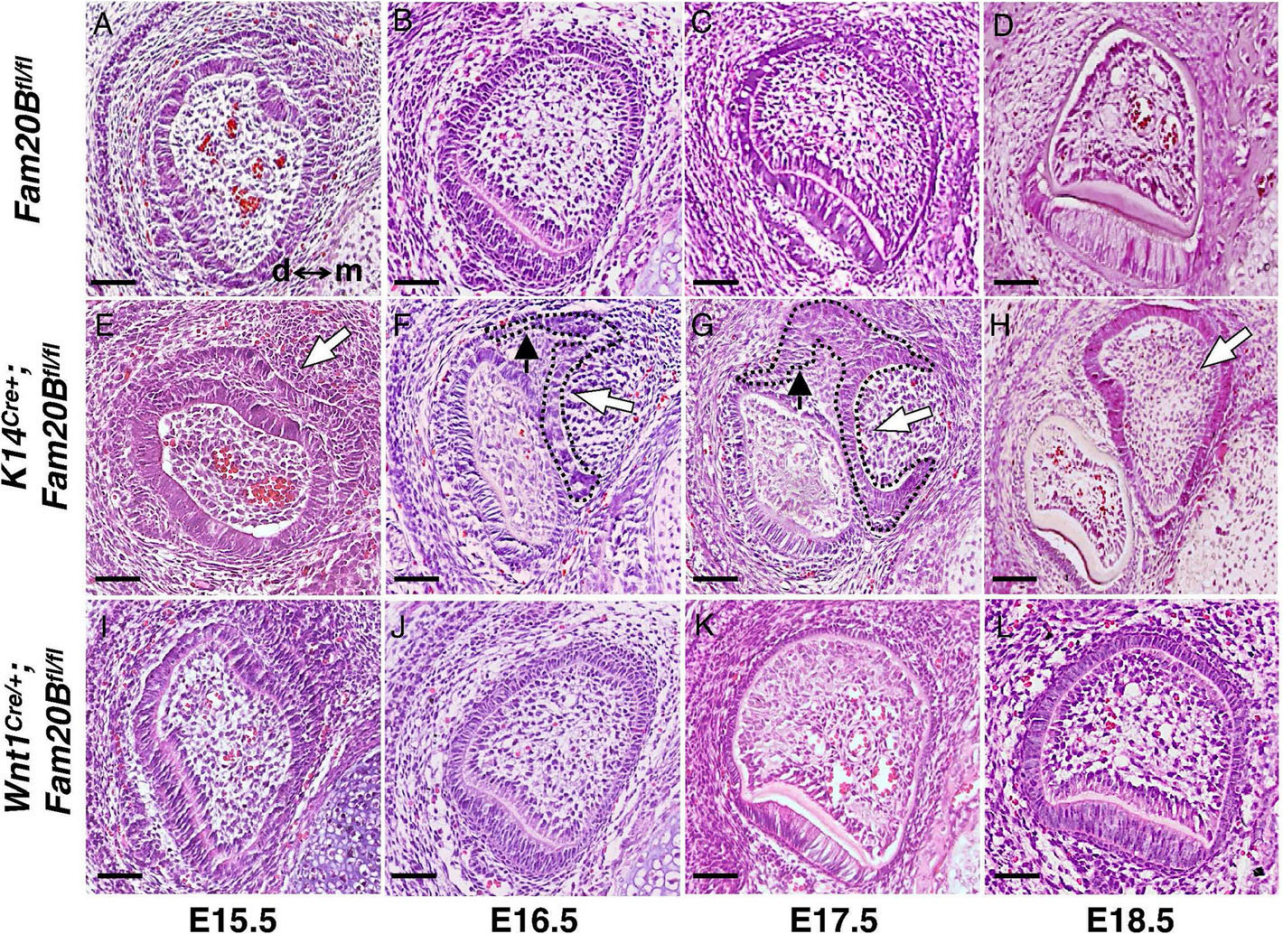
GAGs in the dental epithelium but not in the dental mesenchyme determine the tooth number in mice. Hematoxylin-eosin (H&E) staining on coronal sections of lower incisors from E15.5 to E18.5 mouse embryos. **A**–**D** Native enamel organs of incisors in normal control (*Fam20B*^*fl/fl*^) mice. d↔m indicates the orientation of distal and mesial sides. **E** An ectopic thickening of dental epithelium (white arrow) was identified at the mesial-lingual side of the native enamel organ at E15.5 in *K14*^*Cre/+*^*;Fam20B*^*fl/fl*^ mice. **F**, **G** The ectopic thickening of dental epithelium formed an extended dental lamina (black arrows) at the mesial-lingual side of native enamel organs and developed into a novel enamel organ (white arrows and dashed lines) at the end of the extended lamina. **H** At E18.5, the extended dental lamina disappeared, and the extra enamel organs developed into supernumerary incisors (white arrow) at the mesial-lingual side of native teeth. **I**–**L** In contrast, disrupting GAGs in the dental mesenchyme  did not  cause any extra teeth in the *Wnt1*^*Cre/+*^*;Fam20B*^*fl/fl*^ mice. Scale bars: 50 μm

**Fig. 2:**
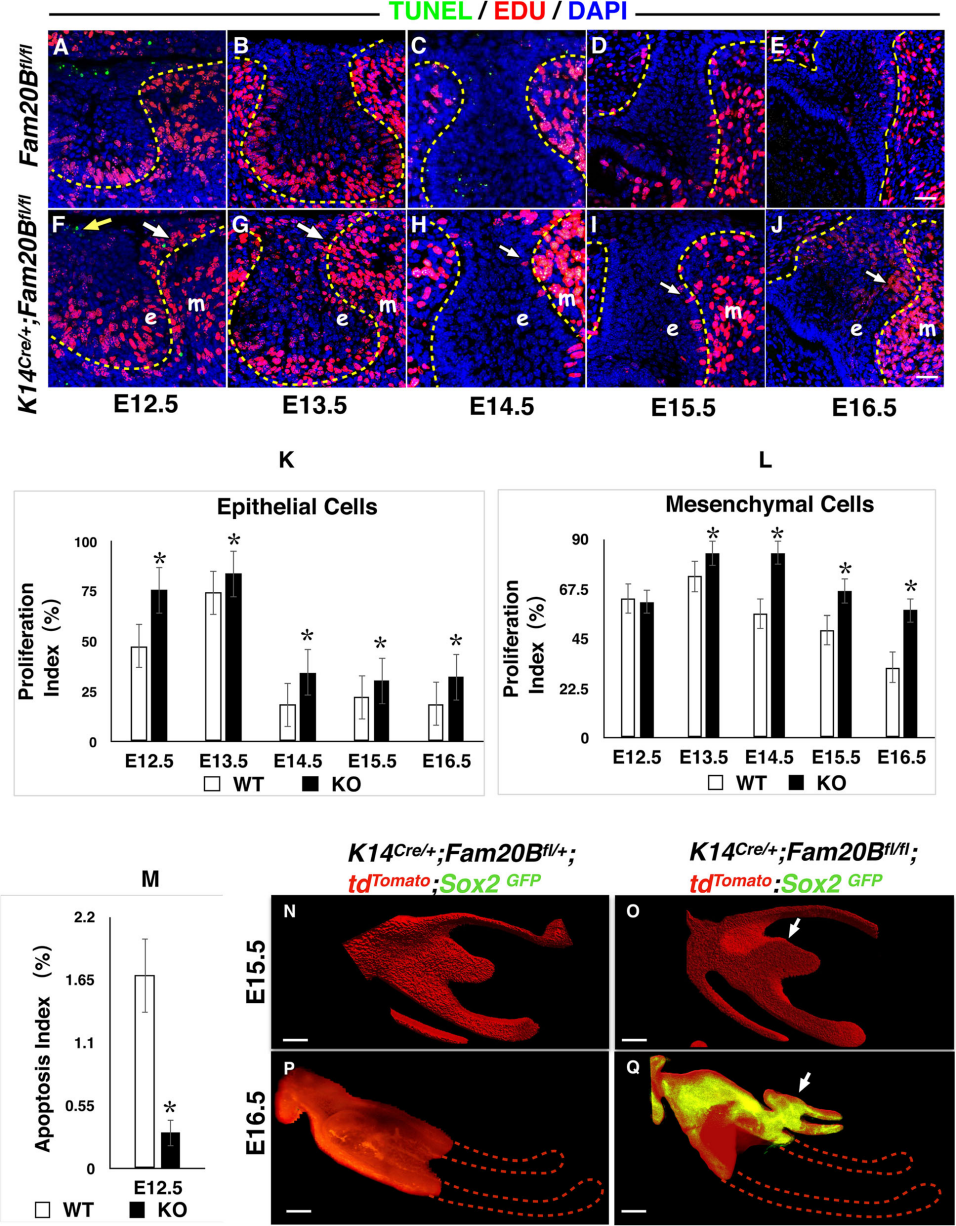
Cell fate change and ectopic renewal of Sox2(+) cells in the dental epithelium of *K14*^*Cre/+*^*;Fam20B*^*fl/fl*^ mice. **A**–**M** GAG deficiency in the dental epithelium led to ectopic over-proliferation and less apoptosis in the native incisors. EdU incorporation analysis showed ectopic over-proliferation (white arrows in **F**–**J**) in the GAG-deficient dental epithelium (e, plotted by dashed lines) and dental mesenchyme (m) at the mesial/lingual side of native incisors in the presumptive location of supernumerary teeth formation starting at E13.5 compared to the normal control mice (2-sample *t* tests, *P* < 0.05). TUNEL assay revealed reduced apoptosis (yellow arrow) in GAG-deficient dental epithelium at E12.5 (2-sample *t* tests, *P* < 0.01). **N**–**Q** Three-dimensional reconstruction of lower incisor confocal images after tissue clearing of mandibles. td^Tomato^ indicates the dental epithelium. GFP indicates the Sox2(+) cells. On the sagittal sections of E15.5 incisors (**N**, **O**), an ectopic thickening of dental epithelium was identified at the lingual side of the native enamel organ in *K14*^*Cre/+*^*;Fam20B*^*fl/fl*^ mice (arrow) compared to the normal control. The slight bulge (white arrow) at the lingual side of the enamel organ in the *Fam20B*-mutant mice indicates the initiation of the supernumerary tooth outgrowth. At E16.5 (**P**, **Q**), Sox2(+) cells showed ectopic renewal in the *Fam20B*-deficient native enamel organ and actively contributed to the development of successive enamel organ (arrow). In contrast, the normal controls had lost *Sox2* expression at this stage. The dashed lines in (**P**) and (**Q**) outlined the remaining parts of the enamel organs that were not able to be displayed due to the working distance of the confocal. Scale bars: 25 μm in (**A**–**J**); 50 μm in (**N**–**Q**)

Inactivating *Fam20B* in the dental epithelium (*K14*^*Cre/+*^; *Fam20B*^*fl/fl*^) led to formation of duplicate incisors ensuing the native ones, while the molar and diastema regions were not affected [12]. Histology and lineage tracing analyses revealed that the duplicate incisors initiate from an outgrowth of the lingual/mesial side dental lamina of the native enamel organ at the late cap stage (~E15.0) (Figs. 1E–H and 2N–Q). Of note is that both the native and duplicate incisors retained the ability of continuous growth and did not show apparent difference in the growth rate.

### Murine tooth number is specifically modulated by the GAGs in the dental epithelium

Supernumerary teeth have been implicated with alterations of the interactions between the dental epithelium and dental mesenchyme [18]. Signaling changes in the dental mesenchyme frequently cause extra tooth formation, such as those in *Gas*^*−/−*^, *Sostdc1*^*−/−*^, *Spry4*^*−/−*^, *Wnt1*^*Cre*^*;Polaris*^*fl/fl*^, and *Osr2*^*−/−*^ mice [19]. To investigate whether GAGs in the dental mesenchyme also play a role in controlling the tooth number in mice, we generated *Wnt1*^*Cre/+*^*;Fam20B*^*fl/fl*^ mice to disrupt GAGs in the neural crest-derived mesenchymal cells. These mice did not show any changes in tooth number (Fig. 1I–L), although several mesenchyme-associated defects occurred as expected in their craniofacial complex [20]. This indicates that the murine tooth number is specifically modulated by the GAGs in the dental epithelium but not in the mesenchyme. Hence, we have focused on exploring its role in the dental epithelium.

### GAGs commit the cell fate of dental epithelium at the initial stage of tooth development

Tooth development in mice initiates from a thickening of the dental epithelium at E10.5 to form a placode. The epithelial placode then invaginates into the dental mesenchyme to form a tooth bud, which further folds into an enamel organ in the following stages. In *K14*^*Cre/+*^*;Fam20B*^*fl/fl*^ mice, the earliest sign of replacement tooth formation appeared at the late cap stage (~E15.5) as an ectopic placodal thickening of the dental epithelium at the mesial-lingual side of the native enamel organ (Fig. 1E–H). TUNEL and EdU incorporation analyses on earlier stages showed overproliferation in both the dental epithelium and dental mesenchyme and reduced apoptosis in the dental epithelium at the lingual/mesial side of native enamel organs starting at ~E12.5 in the *K14*^*Cre/+*^*;Fam20B*^*fl/fl*^ mice (Fig. 2A–M). This suggests that the cell fate of the GAG-deficient dental epithelium had been changed prior to the cap stages. To determine a precise timing of the cell fate change, we employed a Tet-On system to knockout *Fam20B* from the dental epithelium at different embryonic stages. The results demonstrated that replacement tooth formation was induced only if *Fam20B* was inactivated within a time window between E10.5 and E12.5 (Additional File 1: Fig. S1) (Table 1). This implies that the cell fate of primary dental lamina was committed by FAM20B-catalyzed GAGs at the initial stage of tooth development.

**Table 1:**
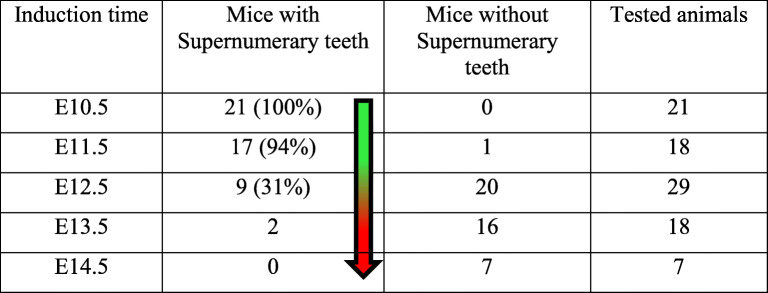
Supernumerary teeth formed when *Fam20B* inactivation was induced between E10.5 and E12.5

### GAGs control murine tooth number by restricting the renewal of Sox2(+) cells in the dental epithelium

*Sox2*-expressing stem/progenitor cells are believed to contribute to the whole enamel organ tissues during tooth development [21, 22]. In cKO (*K14*^*Cre/+*^*;Fam20B*^*fl/fl*^*;Sox2*^*GFP*^) mice, the *Fam20B*-deficient incisors showed progressively increasing expression of ectopic Sox2 at the lingual side of enamel organ from E13.5 to E15.5. In contrast, the Sox2 signal gradually vanished from the lingual side of the enamel organ in control incisors (*K14*^*Cre/+*^*;Fam20B*^*fl/+*^*;Sox2*^*GFP*^) during these stages (Additional File 2: Fig. S2). At E16.5, the normal enamel organ completely lost Sox2(+) expression (Fig. 2P), while the cKO mice showed large amount of Sox2(+) cells in both the lingual side of the native enamel organ, the dental lamina and the enamel organ of the supernumerary teeth (Fig. 2Q). These results suggest that GAG deficiency in the dental epithelium leads to ectopic or extended renewal of Sox2(+) stem/progenitor cells.

Subsequently, we deleted *Sox2* from the dental epithelium of *K14*^*Cre/+*^*;Fam20B*^*fl/fl*^ mice by introducing the *Sox2*^*fl/fl*^ allele to examine the contribution of Sox2(+) progenitor cells to the supernumerary tooth formation in the GAG-deficient teeth. The resultant mice showed partial rescue of the supernumerary tooth phenotype as well as a reduced size of the native teeth (Additional File 3: Fig. S3). To further explore the regulatory input of GAGs on the renewal of Sox2(+) cells, we deleted *Fam20B* from the Sox2(+) population at E11.5 using *Sox2-CreER*, the efficiency of which was validated by crossing with tdTomato indicator (Additional File 4: Fig. S4A). The *Sox2*^*CreER*^*;Fam20B*^*fl/fl*^ mice did not recapitulate the supernumerary tooth phenotype (Additional File 4: Fig. S4B), suggesting that FAM20B-catalyzed GAGs restrict the renewal of Sox2(+) cells in a non-autonomous manner.

### GAGs determine the cell fate of dental epithelium by restricting FGFR2b signaling

**Fig. 3:**
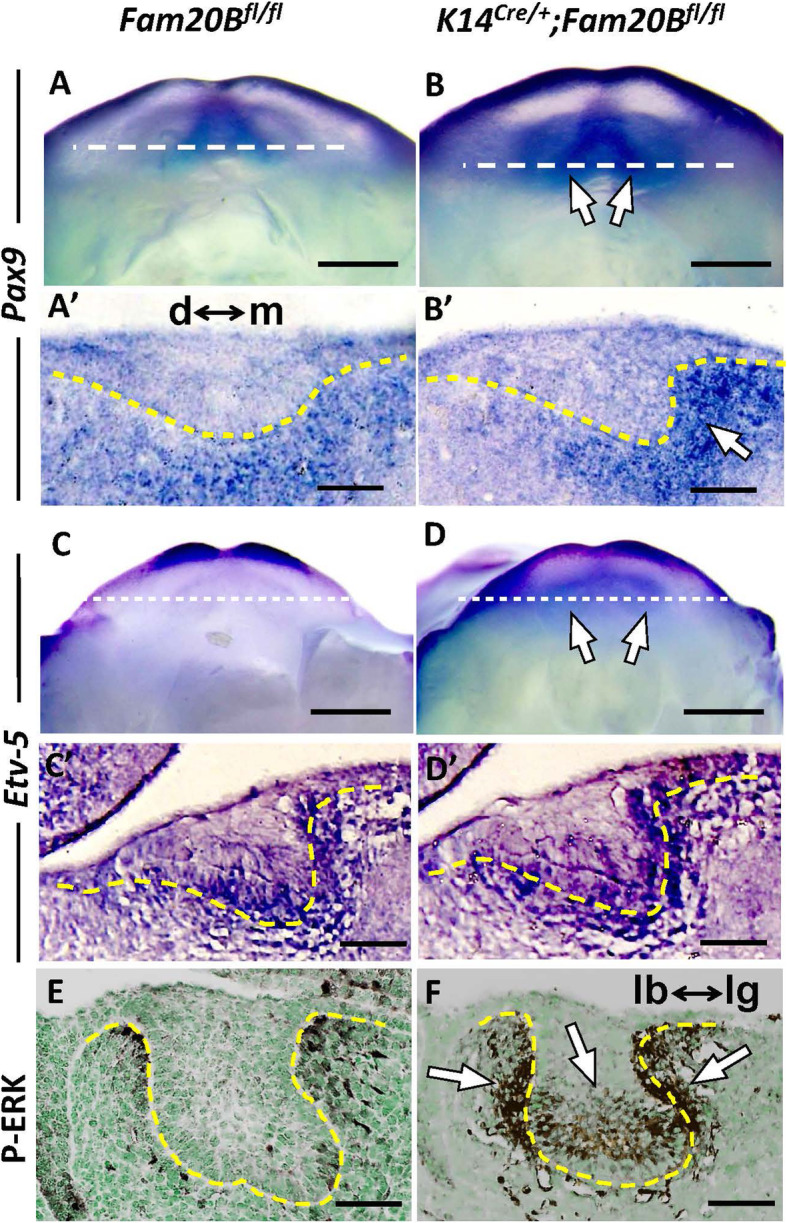
FGF signaling was overactivated in the GAG-deficient incisors. **A**–**D** In situ hybridization of E12.5 whole mount mandibles. **A′**–**D′** Coronal sections through the dashed white lines in (**A**–**D**). The yellow dotted lines indicate the border between the dental epithelium and the dental mesenchyme. d↔m indicates the orientation of distal and mesial sides. In the GAG-deficient incisors, *Pax9* was upregulated at the mesial side of dental mesenchyme (arrow), and *Etv5* showed stronger expression in both the dental epithelium and the dental mesenchyme. **E**, **F** Immunohistochemistry staining of sagittal sections of E13.0 lower incisors showed an upregulation of p-ERK in both the dental epithelium and dental mesenchyme in the *Fam20B*-deficient incisors (white arrows). lb↔lg indicates the orientation of labial and lingual sides. Scale bars: 250 μm in whole mount **A**–**D**; 50 μm in sections **A′**–**D′** and **E**, **F**

**Fig. 4:**
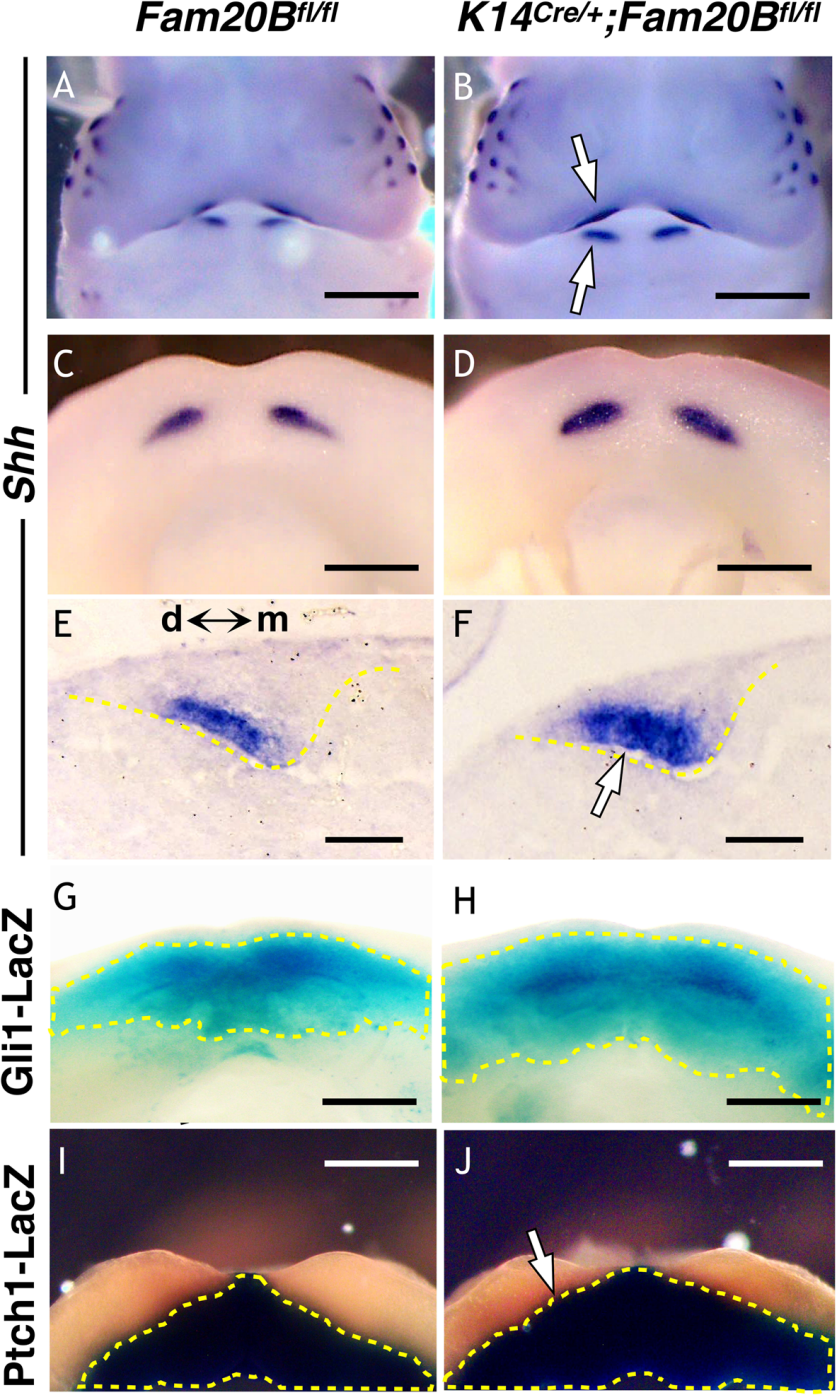
SHH signaling was overactivated in the GAG-deficient incisors. **A**–**D** Whole mount in situ hybridization of E12.0 mouse embryos showed an expanded territory of *Shh* (white arrows) in both upper and lower incisor areas of the *K14*^*Cre/+*^*;Fam20B*^*fl/fl*^ mice compared with the controls (*Fam20B*^*fl/fl*^). Note that *Shh* signal in hair follicles can be used as internal references for the comparison between knockout and control mice. **E**, **F** ISH of *Shh* on the coronal sections of lower incisors showed an expanded expression of *Shh* in the GAG-deficient dental epithelium toward the mesial side (white arrow) compared with controls. **G**–**J** HH downstream markers, Gli1 and Patched1, showed broader reactivity in the dental mesenchyme of *K14*^*Cre/+*^*;Fam20B*^*fl/fl*^ mice, as indicated by Gli1- and Ptched1-LacZ indicators (dashed lines plotted)*.* Scale bars: 500 μm in **A** and **B**; 250 μm in **C**, **D** and **G**–**J**; 50 μm in **E** and **F**

Multiple signaling pathways, such as those mediated by WNTs, FGFs, BMPs, and HH, have been implicated in cell fate commitment in embryonic teeth [23, 24]. To begin to investigate the molecular mechanisms by which GAGs determine the cell fate of dental epithelium, we systematically screened signaling pathways potentially associated with the phenotype at the initial stage of tooth development using immunohistochemistry, in situ hybridization, and signaling reporter lines in mice. We did not detect significant changes in WNT and BMP signaling in the *Fam20B*-mutant incisors (Additional Files 5 and 6: Figs. S5 and S6). However, we identified a hyperactivation of FGF in the *Fam20B*-deficient incisors at E12.5 and E13.0, as indicated by a robust upregulation of *Pax9*, *Etv-5*, and p-ERK in the dental epithelium and/or dental mesenchyme (Fig. 3). In agreement with this, the transcription of *Shh*, a downstream gene of FGF signaling [25], showed an expanded scope of expression at the presumptive location of replacement teeth formation in the GAG-deficient dental epithelium. Accordingly, two SHH downstream markers in the dental mesenchyme, *Gli1* and *Patched1*, showed broader responses to the epithelial HH signaling, as indicated by *Gli1-* and *Ptch1-LacZ* indicator mice (Fig. 4).

Several FGFs and their receptors are expressed in incisors during the critical time window (E11.5-E12.5) identified in the *Fam20B*-mutant mice for supernumerary tooth formation: FGF1, FGF2, and FGF9 are present in the dental epithelium, while their primary receptor, FGFr1c, is expressed in the dental mesenchyme. FGF10 is localized in the dental mesenchyme whereas its receptor, FGFr2b, is exclusively present in the dental epithelium [26] (Additional File 7: Fig. S7). The complementary expression pattern between FGF ligands and their receptors requires FGFs to diffuse to the counterpart location/tissue to perform their functions. We excluded several FGFs and their receptors based on their incongruent expression pattern and timing. For example, FGF8 was excluded for lacking an expression in incisors during this stage, while FGF3 and FGF4 were excluded for their expression timing later than E13.5, etc.

It is well documented that FGFs in the dental epithelium and the dental mesenchyme stimulate one another via a positive feedback loop, which is negatively regulated by Sprouty [27, 28]. Previous studies indicated that inactivation of Sprouty led to supernumerary teeth in both diastema and incisor regions due to epithelium↔mesenchyme bidirectional hyperactivity of FGF, in which FGF10/FGFr2b signaling appeared to play a major role compared with FGF3/FGFr1c [27].

**Fig. 5:**
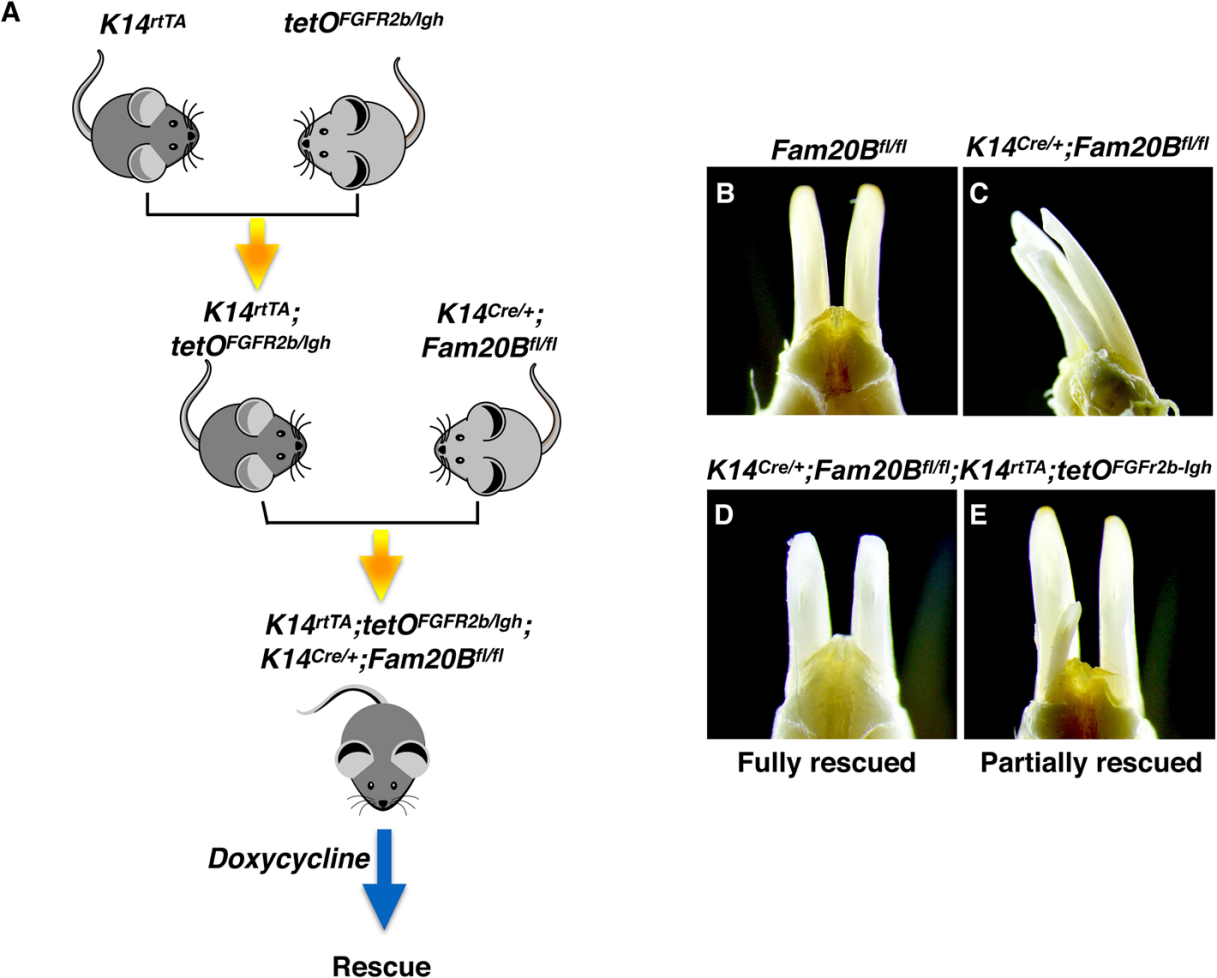
Inhibiting FGFR2b rescued the supernumerary tooth phenotype. **a** The schematic depicts the rescue strategy of supernumerary tooth formation by inhibiting FGFR2b with a dominant-negative FGFR2b inhibitor through a tetO system. The *K14*^*rtTA*^, *tetO*^*FGFr2b/Igh*^, and *K14*^*Cre/+*^*;Fam20B*^*fl/fl*^ mice were crossbred to ultimately generate *K14*^*rtTA*^*;tetO*^*FGFr2b/Igh*^*;K14*^*Cre/+*^*;Fam20B*^*fl/fl*^ mice. Doxycycline was administrated to the resultant mice at 2 mg/kg at designated time points (E10.5–E14.5) to induce the expression of the dominant-negative FGFR2b inhibitor, FGFR2b/Igh, in the GAG-deficient dental epithelium. **b**–**e** Inhibiting FGFR2b in the dental epithelium fully (D) or partially (E) rescued the supernumerary tooth phenotype in the *K14*^*Cre/+*^*;Fam20B*^*fl/fl*^ mice

To determine whether the hyperactivated FGF signaling underlies the cell fate change of the GAG-deficient dental epithelium associated with the extra teeth formation, we employed a Tet-On system to inhibit FGFR2b signaling through overexpressing an FGFR2b inhibitor (dominant-negative FGFR-HFc protein) [29] in the *Fam20B*-deficient dental epithelium. Overexpression of the FGFR2b inhibitor successfully rescued the supernumerary tooth phenotype (Fig. 5) and clearly exhibited a trend whereby earlier inhibition resulted in better rescue effects (Table 2). In agreement with this, inhibiting FGFr2b signaling in the *Fam20B*-deficient dental epithelium also reduced the expression scope of *Shh* back to the normal size (Additional File 8: Fig. S8), indicating that *Shh* is downstream to the FGF hyperactivation that initiates the supernumerary tooth formation. These results collectively indicate that FAM20B-catalyzed GAGs control the cell fate of dental epithelium by confining the FGF10/FGFR2b signaling at the initial stage of tooth formation. The confining effects are most likely associated with FGF10/FGFr2b reactivity/transmission but not their expression, because RNAScope assays of *Fgf10* and *Fgfr2b* did not detect any changes in the *Fam20B*-deficient incisors (Additional File 7: Fig. S7).

**Table 2:**
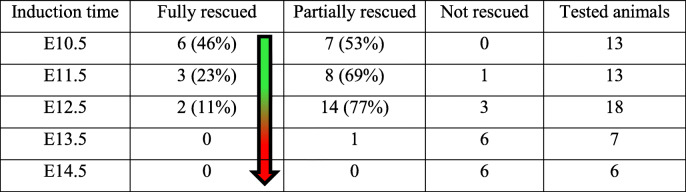
The timing and effects of inhibiting FGFR2b in rescuing the supernumerary tooth formation in *Fam20B*-deficient mice

### GAGs may regulate FGFR2b signaling by confining the diffusion gradient of FGF10

**Fig. 6:**
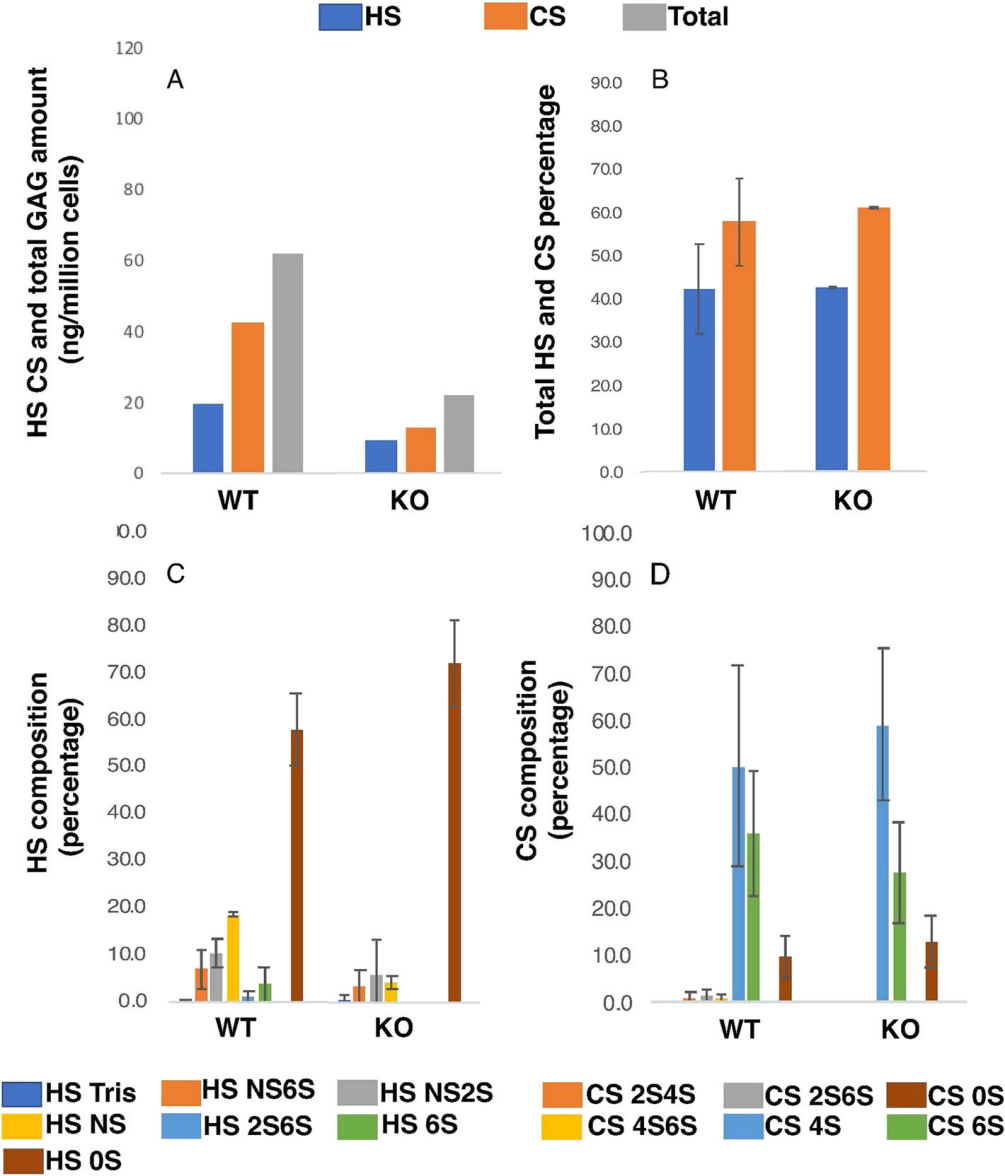
GAG profiling of *Fam20B*-deficient dental epithelium. **A** The amount of HS, CS, and total GAGs were remarkably reduced in the *Fam20B*-deficient dental epithelium compared to the WT (*n* = 1). **B** The HS and CS composition relative to the total GAGs were similar between KO and WT (*n* = 2). **C** Detailed HS composition showed reduced NS and increased 0S in the *Fam20B*-deficient epithelium (*n* = 2). **D** CS composition in the KO did not show apparent differences from the WT (*n* = 2). The analytical error was ~ 3% for these samples

In order to determine how FAM20B loss of function affects GAG assembly in the dental epithelium, we compared the GAG profile of *Fam20B*-deficient dental epithelium with WT using a newly developed GAG profiling method that relies on multiple reaction monitoring liquid chromatography mass spectrometry (MRM-LCMS) [30]. The amount of HS, CS, and total GAGs were remarkably reduced in the *Fam20B*-deficient dental epithelium (Fig. 6A), whereas their total composition did not show apparent changes (Fig. 6B). The HS composition in the *Fam20B*-deficient dental epithelium showed reduced NS and increased 0S (Fig. 6C), while the CS composition in *Fam20B*-deficient dental epithelium did not show apparent differences from the WT (Fig. 6D).

GAGs may regulate growth factor signaling in various manners, including shaping the diffusion gradients and mediating the interactions between the growth factors and their receptors [31]. In a recent study, we revealed that the interactions between FGF10 and heparin are chain-length dependent, and the minimum binding size for the interactions is dp6 [32]. As cells lacking FAM20B cannot extend GAGs beyond the tetrasaccharide linkage and form very short saccharides [10], we estimate that the truncated saccharides of FAM20B-mutant GAGs cannot bind FGFs. In this case, we assayed the interactions between FGF10 and FAM20B non-mutant GAGs (heparin, HS, DiS HS, TriS HS, and CSA) at variable concentrations (0.00, 0.02, 0.08, 0.30, 1.25, and 5.00 μg/ml) in solution culture of BaF3 cells that had been engineered to report FGFR2b reactivity [33]. Heparin served as a positive control in the assays for its known synergistic effects on FGFr2b signaling [34]. Our results showed that none of the tested GAGs, except for the positive control heparin, had significant synergistic or inhibitory effects on FGFR2b signaling (Additional File 9: Figs. S9A and S9B), indicating that GAGs may not restrict FGF10-FGFR2b signaling through modulating ligand-receptor interactions.

**Fig. 7:**
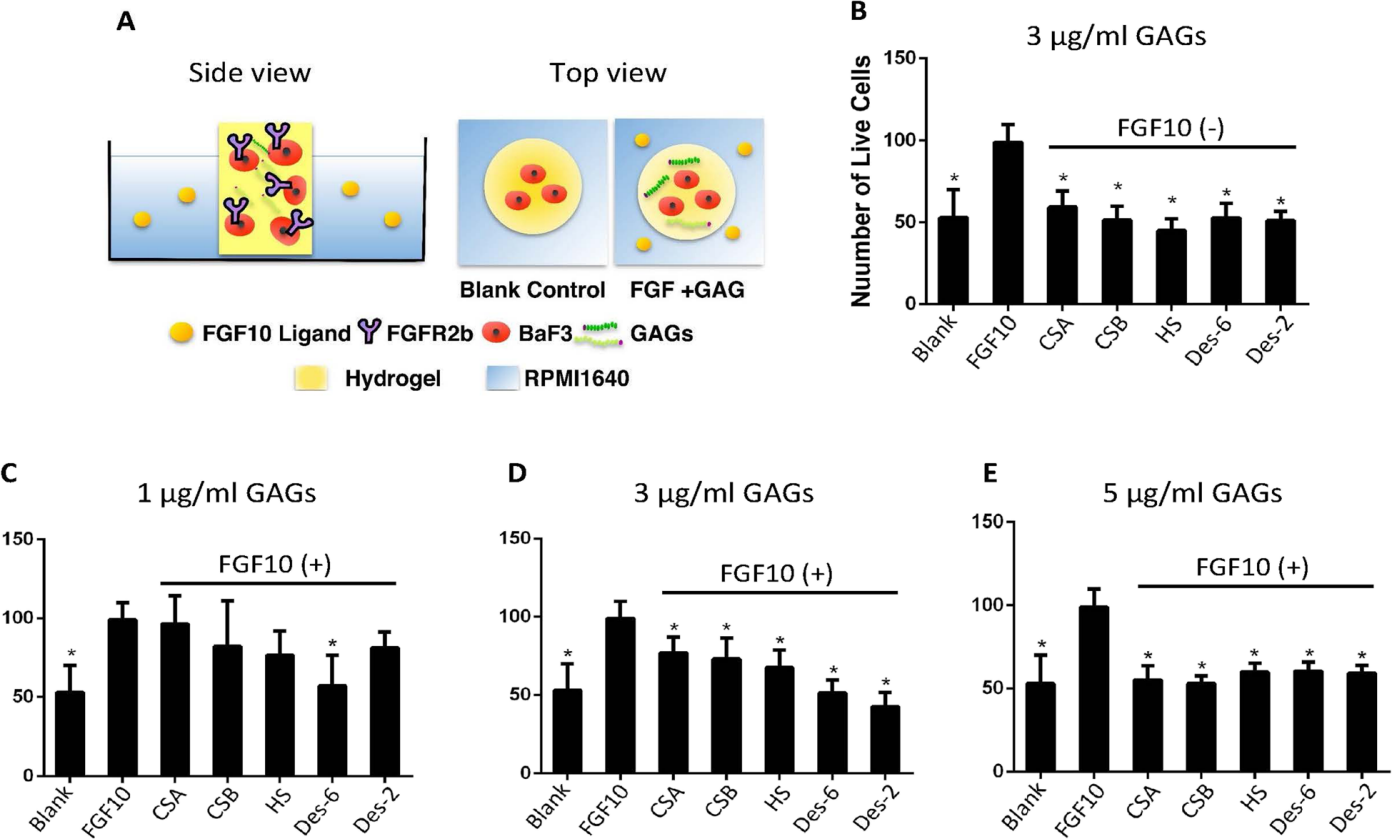
GAGs restrict the diffusion gradient of FGF10. **a** A hydrogel cell culture system was built to mimic the ECM environment. BaF3-FGFR2b cells were embedded in hydrogel with or without GAGs and cultured in RPMI1640 media. Cultures without GAGs in the hydrogel were divided into blank control or FGF10 control according to the presence or absence of 1000 pM FGF10 in the culture medium. The experimental groups were supplemented with 1000 pM FGF10 in the culture medium plus 1 μg/ml, 3 μg/ml, or 5 μg/ml of GAGs in the hydrogel. After 48 h of culture, the FGF activity of each group was calculated based on the viability of BaF3-FGFR2b cells (see Additional File 10: Fig. S10). **b** Cultures with GAGs in the hydrogel without FGF10 in the medium showed no significant differences from the blank control, indicating that GAGs alone did not affect the viability of BaF3 cells. **c**, **d** GAGs at 1 μg/ml and 3 μg/ml concentrations displayed differential config effects on FGF reactivity. **e** GAGs at 5 μg/ml showed nearly equal confining effects on FGF reactivity. The cell viability of each experimental group (named after supplemented GAGs) was compared with FGF control by 2-sample *t* tests. Data are mean ± SD (*n* = 3). *P < 0.05 indicates a significant difference between experimental group and FGF10 Control

Given the complementary expression pattern between FGF10 and FGFr2b, FGF10 needs to diffuse to the dental epithelium to perform its function. We built a hydrogel cell culture system mimicking the ECM environment to test if GAGs (CSA, CSB, HS, Des-2S heparin, and Des-6S heparin) confine the diffusion of FGF10 at certain concentrations (1 μg/ml, 3 μg/ml, and 5 μg/ml) (Fig. 7 and Additional File 10: Fig. S10). We focused on the 2S and 6S since these functional groups are critical in FGF interactions. The requirements of NS and NAc were examined in testing heparin and HS. GAGs at 1 μg/ml and 3 μg/ml displayed differential confining effects on FGF10 diffusion (Fig. 7c, d). In particular, 3 μg/ml GAGs confined FGF10 diffusion in a preference pattern: Des-2 heparin > Des-6 heparin > HS > CSB > CSA (Fig. 7d), and 1 μg/ml GAGs showed a similar preference pattern except for Des-2 heparin displaying a reduced confining effect (Fig. 7c). At 5 μg/ml, all tested GAGs showed almost equally strong inhibition on FGF10 diffusion (Fig. 7e). These data collectively suggest that GAGs differentially confine the diffusion of FGF10 based on their composition and sulfation states in a dose-dependent manner.

### Hyperactivated WNT signaling serves as a relay cue in the GAG-mediated supernumerary tooth formation

**Fig. 8:**
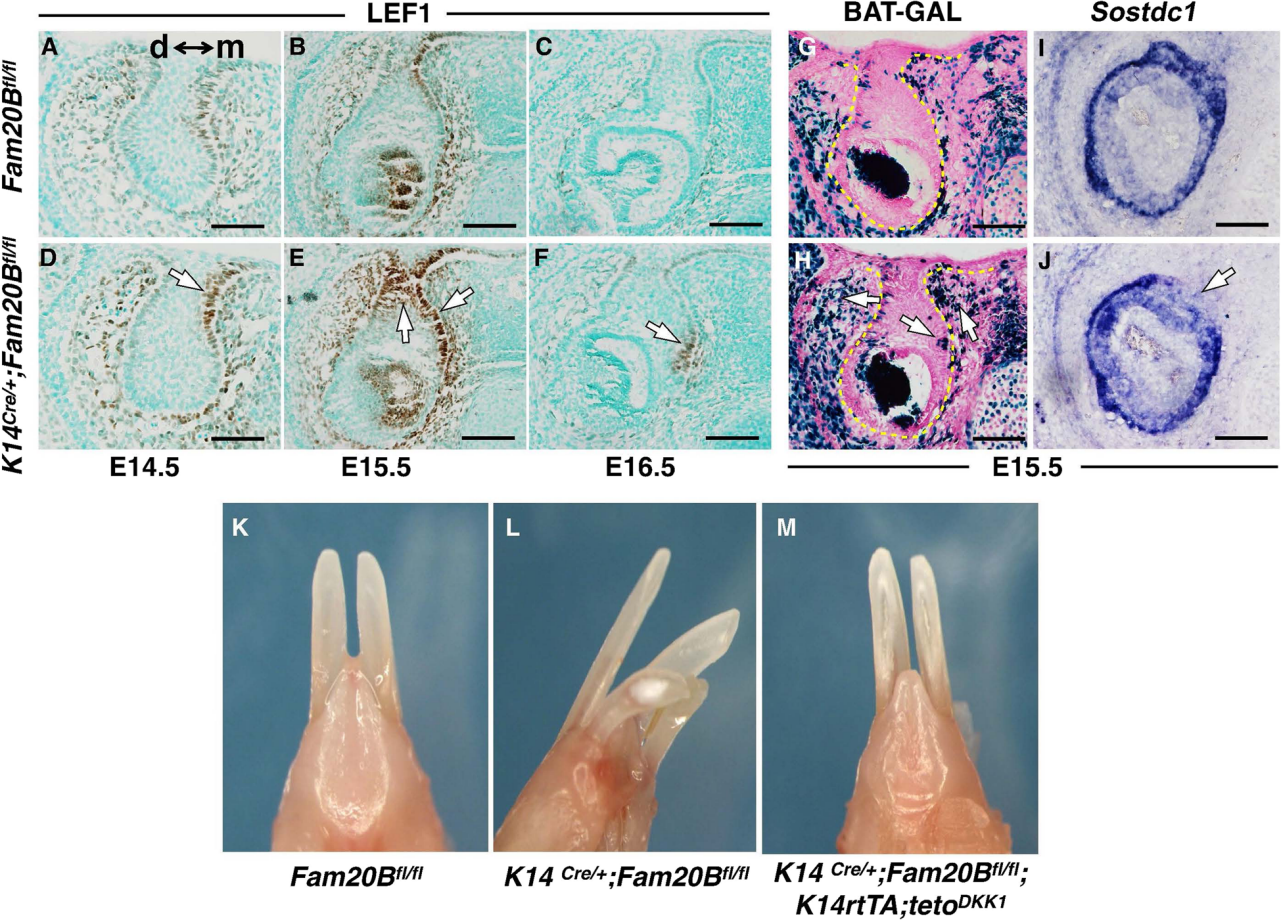
WNT signaling functions as a relay cue facilitating supernumerary tooth formation at late cap stage. **A**–**F** IHC staining of LEF-1, a putative downstream marker of canonical WNT signaling, on the coronal sections of lower incisors at E14.5-E16.5 showed an ectopic overexpression in both the dental epithelium and dental mesenchyme (arrows in **D**, **E** and **F**) at the presumptive location for supernumerary tooth formation (mainly at the mesial-lingual side of native enamel organ) in the *K14*^*Cre/+*^*;Fam20B*^*fl/fl*^ mice compared to the control mice. Note that the LEF1 staining of enamel knots in (**B**) and (**E**) can be used as internal references for comparing the expression levels between control and knockout. d↔m indicates the orientation of distal and mesial sides. **G**, **H** To confirm the upregulation of WNT signaling, we introduced a WNT indicator allele, *BAT-GAL*, into the conditional knockout and control mice. LacZ staining on the coronal sections of E15.5 lower incisors demonstrated more and ectopic WNT activity in the enamel organ and adjacent dental mesenchyme (arrows in **H**) of *K14*^*Cre/+*^*;Fam20B*^*fl/fl*^ mice compared to the control mice. Note that the LacZ staining of enamel knots can be used as internal references for comparing the WNT activity between control and knockout. **I**, **J** Accordingly, the transcription of a WNT inhibitor, Sostdc1, was downregulated in the dental epithelium and adjacent dental follicle at the presumptive location of replacement tooth formation in *K14*^*Cre/+*^*;Fam20B*^*fl/fl*^ mice (arrow in **J**). Scale bars: 200 μm. **K**–**M** To clarify the significance of the overactivated WNT signaling in the supernumerary tooth formation, we overexpressed a WNT inhibitor, DKK1, in the GAG-deficient dental epithelium by introducing *tetO*^*DKK1*^ and *K14*^*rtTA*^ alleles to the *K14*^*Cre/+*^*;Fam20B*^*fl/fl*^ mice. Doxycycline was administrated to the resultant mice at 2 mg/kg at E13.5 to induce DKK1 expression. Overexpression of DKK1 fully rescued the supernumerary tooth phenotype in *K14*^*rtTA*^*;tetO*^*DKK1*^*;K14*^*Cre/+*^*;Fam20B*^*fl/fl*^ mice compared with the normal control (*Fam20B*^*fl/fl*^) and conditional knockout (*K14*^*Cre/+*^*;Fam20B*^*fl/fl*^) mice

Subsequent to the cell fate commitment of the GAG-deficient dental lamina, we identified an ectopic hyperactivation of canonical WNT signaling in the dental epithelium and adjacent dental mesenchyme at the lingual/mesial side of the native enamel organ starting at E14.5 (~ 1 day before the thickening of the extra dental lamina). This is demonstrated by an ectopic expression of LEF-1 (Fig. 8A–F) and BAT-GAL indicator (Fig. 8G, H) compared to the control mice. Conversely, a WNT inhibitor, Sostdc1, was downregulated in the dental follicle at the presumptive location of the supernumerary teeth (i.e., at the lingual/mesial side of native enamel organ) (Fig. 8I, J). The molar regions did not show WNT hyperactivity (data not shown).

We employed a Tet-On system to overexpress DKK1 [35], a WNT inhibitor in the *Fam20B*-deficient dental epithelium at E13.5 to determine the biological significance of the WNT hyperactivation in supernumerary tooth formation. The *Dkk1* transgene fully rescued the tooth phenotype in the *K14*^*Cre/+*^*;Fam20B*^*fl/fl*^ mice (Fig. 8K–M), indicating that the ectopic hyperactivation of WNT signaling is essential for accomplishing the supernumerary tooth formation. However, the upregulation of WNT signaling is not a direct consequence of GAG deficiency, because inactivating *Fam20B* in the dental epithelium at E13.5 (~ 1 day before the overactivation of WNT in the *K14*^*Cre/+*^*;Fam20B*^*fl/fl*^ mice) did not cause supernumerary tooth formation or any WNT activity changes as indicated by *BAT*^*GAL*^ indicator (cKO mice *K14*^*rtTA*^*;tetO*^*Cre*^*;Fam20B*^*fl/fl*^*;BAT*^*GAL*^ versus control mice *K14*^*rtTA*^*;tetO*^*Cre*^*;Fam20B*^*fl/+*^*;BAT*^*GAL*^) (data not shown). These results collectively suggest that the ectopic hyperactivation of WNT is a secondary reaction to the GAG deficiency in the dental epithelium and a relay cue for accomplishing the supernumerary tooth formation.

## Discussion

**Fig. 9:**
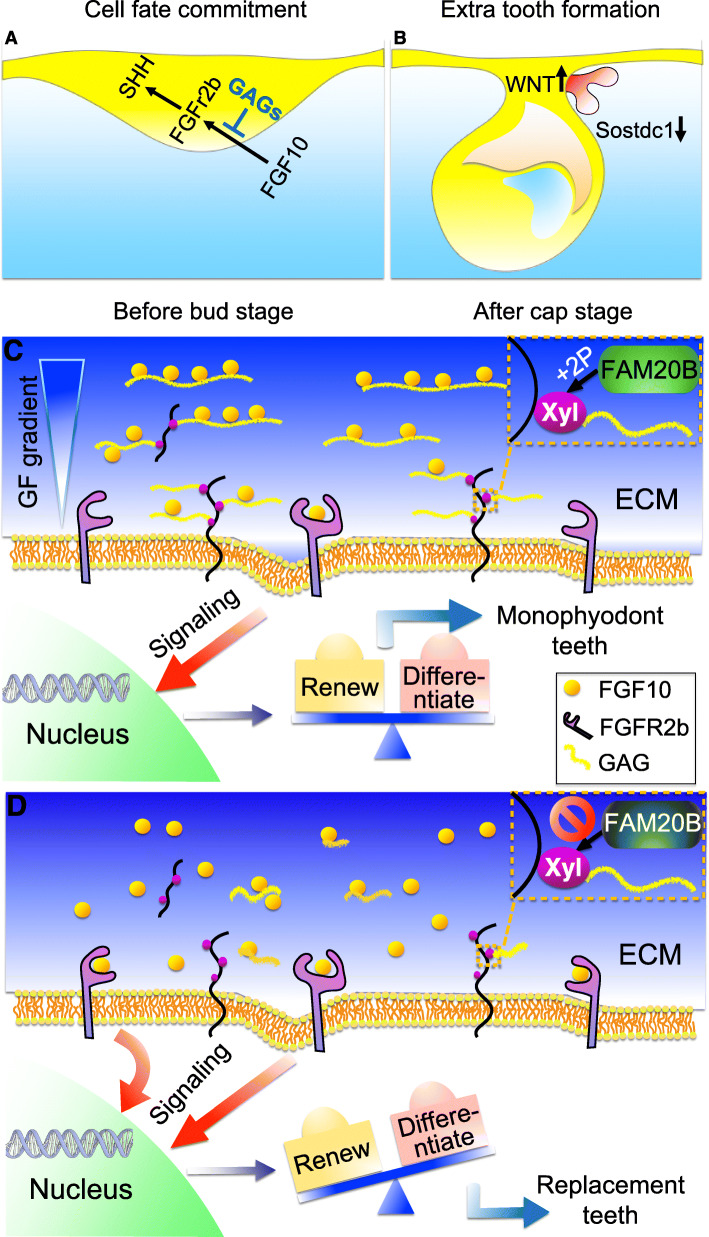
Hypothetic mechanism by which GAGs restrict tooth number in mice. **A** At the initial stage of tooth development, FAM20B-catalyzed GAGs commit the cell fate of dental epithelium by restricting the diffusion gradient of FGF10 from dental mesenchyme into dental epithelium in the process of activating FGFR2b signaling. **B** At late cap stage, overactivated WNT signaling plays as the relay cue to facilitate the supernumerary tooth formation. **C** Under normal condition, FAM20B phosphorylates the xylose in the linkage region of GAGs, which is essential for GAG assembly. GAGs in the ECM and on the cell surface interact with FGF10 ligands to shape a restricted diffusion gradient, which is essential for controlling the FGFR2b-mediated signaling balance between the renewal and differentiation of Sox2(+) stem/progenitor cells in the dental epithelium. **D** Inactivation of *Fam20B* in the dental epithelium leads to GAG deficiency, which facilitates FGF10 diffusion and access to FGFR2b. The upregulated FGFR2b signaling overweighs the renewal of Sox2(+) cells in the dental epithelium, leading to replacement-like tooth formation in monophyodont mice

Interplay between growth factors is involved in the hierarchical and iteratively used  signaling cascades that guidetooth development. These secreted proteins interact with extracellular components to transmit signaling intracellularly. Although accumulating evidence shows that proteoglycans in the extracellular matrix and on the cell surfaces are pivotal signaling regulators for the morphogenesis of multiple organs, their role in tooth development remains poorly understood. In this study, we demonstrate a novel molecular mechanism for how dental epithelium is pre-programmed by GAGs in the control of tooth number in mice. FAM20B-catalyzed GAGs control murine tooth number by committing the cell fate of dental epithelial stem/progenitor cells. This is achieved through restriction of FGFR2b signaling at the initial stage of tooth development. At the cap stages, WNT signaling then relays relevant cues to complete the replacement tooth formation (Fig. 9A, B).

The supernumerary incisors in *Fam20B*-mutant mice initiate from an outgrowth of the lingual/mesial part of the native enamel organs at the late cap stage. This growth pattern is very similar to the supernumerary incisors in *Sostdc1*-mutant mice, which also originate from part of the native teeth, reminiscent of replacement tooth formation [36]. Of note is that *Sostdc1*-mutant mice also develop diastema teeth that are derived from revitalized diastema rudiments [37], and the gene profile change associated with the extra tooth formation is very different from that in the *Fam20B*-mutant mice. Previous studies showed that epithelial stabilization of canonical WNT signaling revitalizes the rudiments in molar and diastema regions [38–41]. In the *Fam20B*-knockout mice, the WNT hyperactivity is confined to the incisor regions starting at the late cap stage and appeared to be a secondary reaction to the hyperactivity of FGF signaling at the initial stage, because removing *Fam20B* from the dental epithelium at the cap stage failed to induce FGF/WNT hyperactivity and supernumerary teeth. These discrepancies suggest intrinsic differences between the development of molars and incisors and illustrate the complexity of the regulatory mechanism for the control of murine tooth number.

*Sox2*-expressing stem/progenitor cells in the dental epithelium are believed to hold the odontogenic potency in different modes of tooth development, including tooth initiation, tooth replacement, and the continuous growth of rodent incisors [19–23, 42]. The role of Sox2(+) cells in the labial cervical loop of murine incisors represents a paradigm of organ renewal derived by adult stem cells [21–23, 43–45]. FGF8 in the stellate reticulum of the cervical loop is believed to maintain the Sox2(+) population in the postnatal incisors [21]. However in embryonic teeth, it remains unclear if a similar molecular mechanism applies to the Sox2(+) population that initiates tooth replacement. Our genetic analyses reveal that the GAG-deficient incisors revived tooth replacement-like capacity but retained the continuous growth, and there were no growth rate differences between the native and replacement incisors. This indicates that the Sox2(+) cells are differentially regulated for tooth renewal and tooth replacement in the cervical loop of postnatal incisors and in the dental epithelium of embryonic teeth.

FGF10-FGFR2b signaling has been associated with stem cell recruitment and maintenance in multiple tissues [46–49]. In embryonic teeth, FGF10 is dominantly present at the initial stage of tooth development [26]. Overexpressing FGF10 in zebrafish produces supernumerary and bicuspid teeth [28]. We found that FAM20B-catalyzed GAGs inhibit tooth replacement of murine incisors by confining the renewal of Sox2(+) cells in the dental epithelium via restricting FGFR2b signaling. This provides genetic evidence that the Sox2(+) cells in the embryonic teeth are differentially regulated by FGFs compared to those in the postnatal teeth. Hence, they modulate different tooth renewal patterns in the processes underlying tooth replacement versus continuous growth in mice.

Although the lineage tracing in this study clearly indicates that Sox2(+) cells contribute to the supernumerary tooth formation in GAG-deficient incisors, deleting *Sox2* from *Fam20B*-deficient dental epithelium only partially rescued the phenotype. Sanz-Navarro et al. [50] also observed mild tooth phenotypes when they removed *Sox2* from Shh(+) population in the dental epithelium. They speculated that it may be related to the mosaic activation of *Shh*-Cre and/or redundancy from other *Sox* members. Together with our data, it appears that *Sox2* is dispensable in the dental epithelium for odontogenesis, although it is an excellent marker for the dental epithelial stem/progenitor cells.

Previous studies have identified undersulfated GAGs on pluripotent embryonic stem cells (ESCs) whereas highly sulfated GAGs on differentiated cells, indicating that the sulfation pattern of GAGs are implicated with the progression of ESCs from self-renewal to a differentiated state [51, 52]. An explanation is that the stemness of ESC*s* is protected from the growth factor signaling by a shield of minimally sulfated GAGs [51]. However, this mechanism may not apply to the stem/progenitor cells in the dental epithelium of mice. The sulfation loss in the dental epithelial GAGs could be a secondary effect that is derived from the dramatic reduction of GAGs. More importantly, the undersulfated GAG remnants in the *Fam20B*-deficient dental epithelium appeared not to shield growth factors, because FGF signaling was upregulated rather than downregulated/abolished.

Accumulating evidence shows that HS/heparin 6-*O*- and 2-*O*-sulfo groups modulate FGF10-mediated epithelial morphogenesis and differentiation (such as those in submandibular and lacrimal glands) by increasing the affinity of FGF10 to FGFR2b, which forms an FGF10-FGFR2b-HS ternary signaling complex to result in diverse biological outcomes [53–55]*.* When *Hs2st* (heparan sulfate 2-*O*-sulfotransferase) and *Hs6st* (heparan sulfate 6-*O*-sulfotransferase 1) were both inactivated, the formation of FGF10-FGFR2b-heparan sulfate complex was disrupted on the cell surface and completely abolished lacrimal gland development [54]. In *Fam20B*-knockout mice, there was no apparent reduction of endogenous HS 6-O- and 2-O-sulfo groups in the Fam20B-deficient dental epithelium. An assumptive disruption of the FGF10-FGFR2b-HS complex in the *Fam20B*-deficient dental epithelium indeed promoted rather than attenuated FGF10-FGFR2b signaling. This in turn promoted the renewal but not differentiation of the Sox2(+) stem/progenitor cells, strongly suggesting an inhibitory role of GAGs on FGF10-FGFR2b signaling. Our results suggest that GAGs in the dental epithelium are unlikely to inhibit FGF10-FGFR2b signaling by serving as co-receptors. Instead, they may regulate the signaling by sequestering FGF10 to restrict the diffusion gradient. The differential regulation of FGF10-FGFR2b signaling between the dental epithelium and other epithelium-derived organs also illustrates that GAG-mediated regulation of growth factors is highly dependent on the biological context.

It is interesting to note that the biological effects of GAG deficiency were very specific in *Fam20B*-deficient dental epithelium despite the broad spectrum of proteins potentially interacting with GAGs. Similar phenomenon has been observed in many other GAG-deficient animal models [17]. The specificity of GAG-growth factor interaction may be derived from a specific polysaccharide sequence, a polysaccharide conformation, an accurate control of enzymatic modification on saccharides, or a dominant presence of growth factors in certain biological context [56]. A prerequisite to answering these questions will require the development of oligosaccharide libraries with systematically varied structures and more sophisticated GAGosome analyses, such as oligosaccharide mapping and sequencing, which are emerging technologies [57].

In summary, this study reveals that FAM20B-catalyzed GAGs determine the monophyodont phenotype in mice by restricting the capacity for renewal of dental epithelial stem/progenitor cells through inhibition of FGFR2b signaling at the initial stage of tooth development. The GAGs interact with FGF ligands to shape a restricted diffusion gradient of FGF10, which maintains a subtle balance between the renewal and differentiation of Sox2(+) cells in the dental epithelium. Disrupting GAG assembly breaks this balance by overactivating FGFR2b signaling, which overweighs the renewal of Sox2(+) cells and initiates the replacement tooth formation in monophyodont mice (Fig. 9C, D).

## Conclusion

In conclusion, this study demonstrates that the FAM20B-catalyzed GAGs control the number of murine teeth by regulating the commitment of dental epithelial stem/progenitor cells through a mechanism involving the restriction of FGFR2b signaling at the initial stage of tooth development. This novel mechanism may also be more broadly relevant for potentiating signaling interactions in other tissues during development and tissue homeostasis.

## Materials and methods

### Animals

All of the animal experiments were carried out according to the protocol approved by the Institutional Animal Care and Use Committee of Texas A&M University College of Dentistry (Dallas, TX, USA) and performed in accordance with the NIH *Guide for the Care and Use of Laboratory Animals*.

*Fam20B*^*flox/flox*^ mice and *TetO*^*Dkk1*^ micewere generated as previously described [12, 35]. *BRE-LacZ* mice were kindly gifted by Dr. Leif Oxburgh (Maine Medical Center Research Institute). Mice purchased from Jackson Laboratory (Bar Harbor, MN, USA): *K14*^*Cre*^ (stock #004782), *Wnt1*^*Cre2*^ (stock #022137) [58], *K14*^*rtTA*^ (stock #008099) [59], *TetO*^*Cre*^ (stock #006224) [60], *TetO*^*FGFr2b/Igh*^*(stock #025672)* [29], *Rosa26*^*tdTomato*^ (stock #007909) [61], *BAT*^*GAL*^ (stock #005317) [62], *Gli1*^*LacZ*^ (stock #008211) [63], *Ptch1*^*LacZ*^ (stock #003081) [64], *Sox2*^*GFP*^ (stock #017592) [65], *Sox2*^*CreER*^ (stock #017593) [65], and *Sox2*^*flox*^ (stock #013093) [66]. Genotyping were carried out as previously described [12] or following Jackson Lab’s instructions.

Pregnant mice were fed with Doxycycline chow (1 g/kg, Bio-Serv, NJ, USA) and water (2 mg/ml, Sigma, St. Louis, MO, USA) at designated time points for 3 consecutive days to induce TetO transgene expression. Tamoxifen (T5648, Sigma, St. Louis, MO, USA) was injected to pregnant mice intraperitoneally at 50 mg/kg at designated time points for 3 consecutive days to induce CreER expression.

### Tissue preparation

Embryonic stage was determined according to the vaginal plug (day 0.5) and confirmed by morphological criteria. Mouse embryos of desired stages were harvested in diethyl pyrocarbonate (DEPC)-treated Dulbecco’s phosphate-buffered saline (PBS). Dissected heads from the embryos were fixed with 4% paraformaldehyde (PFA) in 0.1% diethyl pyrocarbonate(DEPC)-treated PBS at 4 °C overnight and decalcified in 0.1% DEPC-treated 15% EDTA (pH 7.4) at 4 °C for 1 to 2 days as needed, then dehydrated in a serial gradient of ethyl alcohol solutions followed by paraffin embedding.

### Tissue clearing and 3D imaging

Mandibles for 3D-image reconstruction were collected from 6 embryos of KO or control mice at each desired time point. Tissue clearing was performed on the mandibles as previously described [67]. Fluorescent images were acquired with Zeiss LSM780 two-photon microscopy (Visible laser lines: 488,633 nm). Image processing and 3D rendering were performed with Imaris 9.0 (Bitplane) as previously described [67].

### Immunohistochemistry (IHC)

Immunohistochemistry staining was performed on 4-μm-thick coronal sections prepared from 6 mandibles of KO or control mice using a DAB substrate kit (Vector Laboratories, Burlingame, CA, USA) following the manufacturer’s instruction. The primary antibodies used for immunohistochemistry are: anti-phospho-ERK (9101, 1:200, Cell Signaling, Danvers, MA, USA), anti-Lef1 (C12A5, 1:200, Cell signaling, Danver, MA, USA), anti-β-catenin (sc-7963, Santa Cruz), anti-Sox2 (ab97959, Abcam, Cambridge, MA, USA), and anti-phospho-SMAD1/5 (9516, Cell Signaling). Methyl green was used for counterstaining.

### In situ hybridization (ISH)

ISH was performed following previously descripted protocols [68, 69]. For section ISH, the paraffin-embedded samples were prepared as 10-μm-thick serial sections. For whole-mount ISH, the properly fixed embryos were bleached with 3% H_2_O_2_ followed by dehydration in methanol. Plasmids containing the cDNAs of mouse *Shh*, *Pax9*, *Msx-1*, *Sostdc1*, and *Wnt5a*, were linearized with appropriate restriction enzymes. The cDNA of *Etv-5* was generated by RT-PCR using the total RNA extracted from E13.5 mouse embryos and designed primers (Forward: AGTGGCCGCTCAGGAGTA; Reverse: AGCTATTTAGGTGACACTATAGACAGTAATCTCGGGG CTCCT). The sense and antisense probes were synthesized using an RNA Labeling Kit (Roche; Indianapolis, IN). The probes were detected by an enzyme-linked immunoassay with an anti-DIG-AP antibody conjugate (Roche, Indianapolis, IN, USA) and stained with BM Purple (Roche) for positive signals. The DIG-labeled sense probes were used in place of the antisense probes in the negative control experiments. The results were examined and photographed using an Olympus RX43 upright microscope and an SZX16 stereo microscope (Olympus, Waltham, MA, USA) connected with a DP27 imaging system (Olympus).

### RNAScope and quantitation

RNAScope was performed using the RNAscope 2.5 HD Brown Reagent Kit (322300, Advanced Cell Diagnostics, Neward, CA, USA) on 5-μm FFPE tissue sections prepared from 6 mandibles of KO or control mice according to the manufacturer’s instructions. Slides were baked for 1 h at 60 °C prior to use. After de-paraffinization and dehydration, the tissues were air dried and treated with peroxidase blocker before boiling at 100–104 °C in target retrieval reagents for 15 min. Protease was then applied for 30 min at 40 °C. Target probes *Fgf10*, *Fgfr2b*, and *Fgf9* (446371, 806301, 499811, Advanced Cell Diagnostics) were hybridized for 2 h at 40 °C, followed by a series of signal amplification and washing steps. All hybridizations at 40 °C were performed in a HybEZ Hybridization System. RNA staining signal was identified by DAB as brown chromogenic dots. Following the RNAscope assay, samples were counterstained for 2 min with hematoxylin. Each sample was quality controlled for RNA integrity with a probe specific to the housekeeping gene cyclophilin B (PPIB); only samples with an average of > 4 dots per cell were included for analysis. Negative control background staining was evaluated using a probe specific to the bacterial dapB gene; only samples with an average of < 1 dot per 10 cells were included for analysis. Bright field images were acquired by Olympus CKX41 inverted microscope using a × 40 objective.

For semi-quantitation analysis, the RNAscope signal is scored on the basis of number of dots per cell as follows: 0 = 0 dot/cell, 1 = 1–3 dots/cell, 2 = 4–10 dots/cell, 3 = 10–15 dots/cell, and 4 = > 15 dots/cell with > 10% of dots in clusters. To evaluate heterogeneity in marker expression, *H*-score analysis is performed. The *H*-score is calculated by adding up the percentage of cells in each scoring category multiplied by the corresponding score, so the scores are on a scale of 0–400. The RNAscope signal area proportion of each probe is quantified by Image J based on × 20 tooth bud images (250 × 180 μm).

### Cell proliferation assay (EdU) and TUNEL staining

Timed pregnant mice were injected intraperitoneally with EdU at 15 μg/kg in PBS (C10352, Invitrogen, Carlsbad, CA, USA). After 1 h of injection, embryo heads were collected and processed for paraffin embedding and section. EdU incorporation was detected on 5-μm-thick paraffin sections using a Click-iT Kit (Invitrogen) following the manufacturer’s protocol. Apoptotic cells were identified on the sections by TUNEL staining using an ApopTag Plus In Situ Apoptosis Fluorescein Detection Kit (S7111, Millipore, Burlington, MA, USA) according to the manufacturer’s instruction. DAPI was used as counterstaining. Mounted sections were examined and photographed using an SP5 confocal microscope (Leica, Buffalo Grove, IL, USA).

### X-Gal staining

The embryos for whole-mount X-Gal staining were fixed with 0.2% glutaraldehyde in PBS at 4 °C for 30 min. After three wash in 0.005% NP-40 and 0.01% sodium deoxycholate, the embryos were incubated in staining solution (5 mM potassium ferrocyanide and potassium ferricyanide, 2 mM MgCl_2_, 0.4% X-Gal in *dimethylformamide*) at 37 °C for 3–24 h, followed by post-fixation with 4% paraformaldehyde (PFA) in PBS at room temperature for 1 h. Embryos for cryosection were fixed in 4% PFA for 1–2 h at room temp and dehydrated in 30% sucrose at 4 °C overnight, then embedded in OCT for cryosection. X-Gal staining was performed on the cryosections, and nuclear fast red was used for counterstaining.

### GAG profiling

GAG profile of E11.5 dental epithelium in WT and *Fam20B*-deficient (KO) mice were characterized regarding the GAG type, amount, sulfation, and disaccharide composition using a recently developed method MRM-LCMS (multiple reaction monitoring liquid chromatography mass spectrometry) [70]. Briefly, the dental epithelium of lower incisors was isolated from the mandibles of E11.5 KO and WT embryos after dispase digestion (1.8 U/ml in Ca- and Mg-free PBS, Gibco) at 37 °C for 30 min. The epithelium pooled from 6 embryos of each group were lysed in digestion buffer (50 mM ammonium acetate, 2 mM calcium chloride) and digested by cocktail of GAG-lyases (heparin lyase I, II, III, and chondroitin lyase ABC (10 mU each), then placed in 37 °C incubator overnight. The resulting disaccharides were recovered by centrifugal filtration, labeled with 2-aminoacridone (AMAC), and analyzed by liquid chromatography mass spectrometry (LC-MS/MS, Thermo Inc.) running at multiple reaction monitoring mode. The separation was carried out with an Agilent 1200 HPLC separation system on an Agilent Poroshell 120 ECC18 column (3.0 × 150 mm, 2.7 μm, Agilent, USA) at 45 °C. The analytical error for GAG profiling was < 3%.

### Cell culture

The BaF3 cells used in this study were engineered to stably express FGFR2b [71]. The cells were maintained in RPMI 1640 culture media (Gibco Life Science, Gaithersburg, MD, USA) supplemented with 10% newborn calf serum (Gibco), 0.5 ng/ml murine recombinant interleukin-3 (Gibco), 2 mM l-glutamine, penicillin/streptomycin (P/S), and 50 nM β-mercaptoethanol (Gibco). The cells were treated with G418 (600 μg/ml, Gibco) for 2 weeks before being used for the subsequent assays.

#### Cell proliferation assay

BaF3-FGFR2b cells (4 × 10^4^ ) were seeded in each well of 96-well plates in culture medium without interleukin-3. The culture medium was supplemented with or without 1.5 μg/ml heparin (as co-receptor for FGF signaling) for the assays of inhibitory or synergistic effects of GAGs on FGF10-FGFR2b reactivity. FGF10 (1000 pM) in 10% BSA and 0–5 μg/ml (0, 0.02, 0.08, 0.30, 1.25, and 5.00 μg/ml) of HS, DiS HS, TriS HS, CSA or heparin were added to each well in duplicates for each group. The cells were incubated at 37 °C for 36–48 h before being assayed for cell number/viability with the CCK-8 kit (Sigma) following the manufacturer’s instruction. Two-way ANOVA shows that heparin is different from all other GAGs, *p* < 0.001. DiS HS was synthesized from *N*-sulfo heparosan with modification using C5-epimerase and 2-*O*-sulfotransferase (2OST) following our previously reported procedures [72]. TriS HS (NS2S6S) was synthesized from *N*-sulfo heparosan with subsequent modification with C5-epimerase, 2-*O*-sulfotransferase, and 6-*O*-sulfotransferases (6OST1/6OST3) [72].

#### 3D hydrogel cell culture

A hydrogel cell culture system was used to mimic the extracellular matrix context in the dental epithelium (Fig. 7a). Briefly, 1.0 μg/ml, 3.0 μg/ml, or 5.0 μg/ml of each GAGs (CSA, CSB, HS, Des-2, and Des-6) in 10 × RPMI1640 and 2.0 × 10^4^ /ml BaF3-FGFR2b cells were mixed with premade collagen solution (2 mg/ml, C4243, Sigma). In each well of 24-well plates, 500 μl of such mixture was dispensed and incubated at 37 °C for 1 h to allow gelation. The hydrogel cylinders were then maintained in RPMI culture media supplemented with 100 ng/ml recombinant human FGF-10 (Invitrogen) for further analyses.

#### Cell viability assay

Hydrogel was collected for cell viability assay on day 2 of culture using a Live/Dead Viability Kit (Invitrogen). After washing in PBS for 5 min, the hydrogel was incubated in 2 μM calcein AM and 4 μM ethidium homodimer-1 prepared in PBS in the dark at 37 °C for 40 min, then washed in PBS for imaging. Fluorescent images were obtained with a Zeiss LSM 880 two-photon microscopy (Visible laser lines: 406 and 488 nm). The number of live cells in each sample was counted in 3D hydrogel chips reconstructed from 6 randomly selected areas using Imaris 9.0 (Bitplane) and subjected to statistical analysis.

### Statistics

The data was expressed as mean ± SD of at least 6 determinations in all experiments unless otherwise indicated. We used 2-sample *t* tests to evaluate the pairs of samples and independent-samples *T* test for the independent samples. Before performing *t* test, normal distribution was verified by Levene’s test using SPSS (IBM, NY, USA). Two-way ANOVA was used to compare multiple groups. A *P* value of < 0.05 was considered to indicate statistically significant differences.

## Supplementary information

**Additional file 1: Figure S1.** GAGs commit the cell fate of dental epithelium at the initial stage of tooth development. **A***K14*^*rtTA*^, *tetO*^*Cre*^ and *Fam20B-flox* mice were crossbred to ultimately generate *K14*^*rtTA*^*;tetO*^*Cre*^*;Fam20B*^*fl/fl*^ mice. **B** Doxycycline (Dox) was administrated to the resultant mice at 2 mg/kg at designated time points (E10.5-E14.5) to induce Cre-loxP mediated *Fam20B* deletion from the dental epithelium: the transactivator rtTA driven by epithelium-specific *Keratin-14* promoter recognizes *tetO* sequences in the presence of doxycycline and initiates the transcription of CRE recombinase, thereby deleting *Fam20B* allele between two loxP sites.

**Additional file 2: Figure S2.** The dynamics of Sox2 expression during supernumerary incisor formation. IHC staining of Sox2 was performed on sagittal sections of lower incisors. lb↔lg indicates the orientation of labial and lingual sides. **A, B** At E12.5, the *Fam20b*-deficient dental epithelium started showing more Sox2 expression than normal. **C, D** At E13.5, the control incisors showed reduced expression of Sox2 in the dental epithelium, while the *Fam20b*-deficient dental epithelium had strong expression of Sox2 in the lingual side of the enamel organ (arrow). **E-H** At E14.5 and E15.5, the control incisors gradually lost Sox2 expression from the lingual side of enamel organ. In contrast, the *Fam20B*-deficient incisors showed strong ectopic Sox2 expression at the lingual side of enamel organ (arrows). Scale bars, 50 μm in A and B, 100 μm in C and D, 200 μm in E and F, 400 μm in G and H.

**Additional file 3: Figure S3.** Inactivation of *Sox2* from the dental epithelium partially rescued the supernumerary tooth phenotype in *K14*^*Cre/+*^*;Fam20B*^*fl/fl*^ mice. We introduced *Sox2*^*fl/fl*^ allele into *K14*^*Cre/+*^*;Fam20B*^*fl/fl*^ mice to inactivate both *Sox2* and *Fam20B* in the dental epithelium. The double knockout (*K14*^*Cre/+*^*;Fam20B*^*fl/fl*^*;Sox2*^*fl/fl*^) mice showed reduced number and size of supernumerary teeth (black arrows) compared with *Fam20B* single-knockout (*K14*^*Cre/+*^*;Fam20B*^*fl/fl*^*; Sox2*^*fl/+*^) mice. The native teeth lacking *Sox2* allele (*K14*^*Cre/+*^*;Fam20B*^*fl/+*^*;Sox2*^*fl/fl*^) displayed a smaller size than normal (white arrows).

**Additional file 4: Figure S4.** FAM20B-catalyzed GAGs regulate the homeostasis of *Sox2*^+^ cells in a non-autonomous manner. **A** To validate the efficiency of the Sox2-CreER, we crossbred the *CreER* line with Rosa26-tdTomato indicator mice and induced the Cre expression with single I.P. injection of tamoxifen at E11.5. The embryos were collected at E12.5 and subjected to cryosection for fluorescence assay. On the same cryosections, Sox2-expressing cells were labeled by immunofluorescence using anti-Sox2 antibody and EGFP-conjugated secondary antibody. The Cre activity indicated by Tomato fluorescence (red) was strongly present in the dental epithelium (arrows) and oral epithelium, as well as nasal mucosa and palatal epithelium. The antibody-labeled Sox2-expressing cells (green) mostly overlapped with CreER active cells (red) and showed yellow on the merged channel. CreER-active cells showed overall broader range than the antibody-labeled Sox2(+) cells in the dental epithelium (especially in the distal side) and nasal mucosa, indicating that the efficiency of Sox2-CreER was strong enough for deleting floxed alleles from the Sox2-expressing cells. n, nose; p, palate; m, mandible. **B** To determine the regulatory manner of GAGs on Sox2(+) cell homeostasis, we inactivated *Fam20B* from Sox2(+) lineage using *Sox2-CreER*. Tamoxifen was administered to mice by *i.p.* injection at E11.5 and E12.0. *Sox2*^*CreER*^*;Fam20B*^*fl/fl*^ mice did not recapitulate the replacement tooth phenotype, suggesting that GAGs regulate the homeostasis of Sox2(+) cells in a non-autonomous manner.

**Additional file 5: Figure S5.** WNT signaling was not changed in the *Fam20B*-deficient incisors at the early stage of tooth development. **A, B** Immunohistochemistry staining of LEF1 on the coronal sections of lower incisors showed no differences between the *Fam20B*-deficient and control incisors at E12.5. d↔m indicates the orientation of distal and mesial sides. **C, D** Whole-mount staining of BAT-Gal indicator on E12.5 mandibles showed no differences between the *Fam20B*-mutants and controls. The yellow dotted lines plotted the areas of LacZ positive staining. **E, F** Immunohistochemistry staining of β-Catenin on the coronal sections of lower incisors showed no differences between the *Fam20B*-deficient and control incisors at E12.5. **G, H** In situ hybridyzation staining of *Wnt5a* on the coronal sections of lower incisors showed no differences between the *Fam20B*-deficient and control incisors at E13.5. The yellow dotted lines in E-H indicate the boarder line between the dental epithelium and the dental mesenchyme. Scale bars, 100 μm.

**Additional file 6: Figure S6.** BMP signaling was not changed in the *Fam20B*-deficient incisors at the early stage of tooth development. **A-D** In situ hybridyzation staining of *Msx-1* on the coronal sections of lower incisors showed no differences between the *Fam20B*-deficient and control incisors at E12.5 and E13.5. d↔m indicates the orientation of distal and mesial sides. The yellow dotted lines indicate the boarder line between the dental epithelium and the dental mesenchyme. **E, F** Whole-mount staining of BRE-LacZ indicator on E12.5 mandibles showed no differences between the incisors of *Fam20B*-mutants and controls. The yellow dotted lines plotted the areas of LacZ positive staining in the lower incisors. **G, H** Whole-mount ISH staining of *Bmp4* on E12.5 mandibles showed no differences between the incisors of *Fam20B*-mutants and controls. **I, J** Immunohistochemistry staining of p-SMAD1/5 on the coronal sections of lower incisors showed no differences between the *Fam20B*-deficient and control incisors at E12.5. **K, L** In situ hybridyzation staining of *Sostdc-1* on the coronal sections of lower incisors showed no differences between the *Fam20B*-deficient and control incisors at E12.5. Scale bars, 100 μm.

**Additional file 7: Figure S7**. The transcriptional expression of *Fgf10*, *Fgfr2b* and *Fgf9* was not changed in the *Fam20B*-deficient incisors at the early stage of tooth development. **A- F** RNAScope staining of *Fgf10*, *Fgfr2b* and *Fgf9* on the coronal sections of lower incisors showed no differences between the *Fam20B*-deficient and control incisors at E12.5. The dotted lines indicate the boarder line between the dental epithelium and the dental mesenchyme. **G** Semi-quantitative analysis of RNAScope results showed no significant differences in the transcriptional expression of *Fgf10*, *Fgfr2b* and *Fgf9* between the *Fam20B*-deficient (KO) and control (WT) incisors. Scale bars, 100 μm in A and B; 50 μm in C-F.

**Additional file 8: Figure S8.** Inhibition of Fgfr2b in the *Fam20B*-deficient dental epithelium reduced the expanded expression scope of *Shh* back to the normal size. Scale bars, 250 μm.

**Additional file 9: Figures S9A-S9B.** Fig. S9A-[GAGs did not show synergistic effects on FGF10-FGFR2b signaling]. Fig. S9B-[GAGs did not show inhibitory effects on FGF10-FGFR2b signaling]. HS and CS did not show significant synergistic or inhibitory effects on FGF10-FGFR2b signaling in BaF3 cells. **A** BaF3 cells expressing FGFR2b were cultured in RPMI 1640 media supplemented with 1000 pM FGF10 and 0–5 ng/ml GAGs (HS/HS2S/HS6S/CSA/heparin) for 45 h. Heparin (positive control) showed significant synergistic effects on FGFR2b signaling (*P* < 0.001), while HS/HS2S/HS6S/CSA groups did not show any significant synergistic effects (*P* > 0.05). **B** BaF3-FGFR2b cells were cultured in RPMI 1640 media supplemented with 1000 pM FGF10 and 1.5 μg/ml heparin to boost the baseline of FGFR2b signaling activity (indicated by cell viability OD value). HS/CSA/CSC/CSE/LMW heparin (0–5 ng/ml) supplemented to the culture media did not show any inhibitory effects on FGFR2b signaling (P > 0.05). Two-way ANOVA was used to evaluate the differences among groups.

**Additional file 10: Figure S10.** Live image of BaF3 cells in the hydrogel. After 48 h of culture, BaF3-FGFR2b cells were stained with Live/Dead Staining. Calcein AM in Green represents live cells. DAPI was used for counter staining. Live images of the cells in hydrogel were acquired by confocal microscopy and reconstructed with Imaris 9.0. The number of live cells in each sample was counted from 3D hydrogel chips reconstructed from 6 randomly selected areas for statistical analysis.

## Data Availability

All data generated during this study are included in this published article and its additional files. Raw data and materials will be available at request.
