# Supplementary figures and images for: FAM20B-catalyzed glycosaminoglycans control murine tooth number by restricting FGFR2b signaling
Source: BMC Biol. 2020 Jul 14;18:87. doi: 10.1186/s12915-020-00813-4 (PMC7359594; doi:10.1186/s12915-020-00813-4)

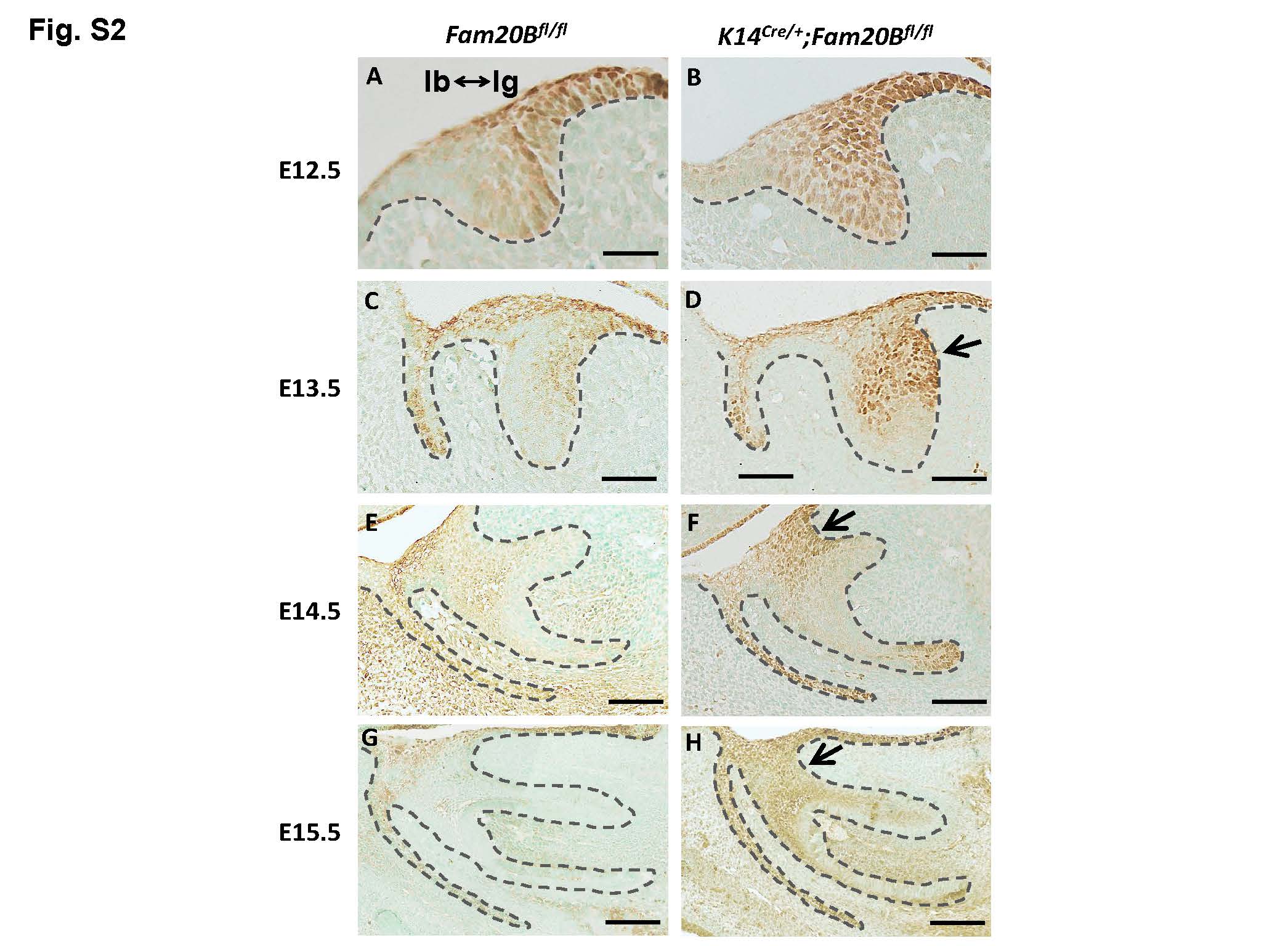

Supplement: Supplementary file 2 — Additional file 2: Figure S2. The dynamics of Sox2 expression during supernumerary incisor formation. IHC staining of Sox2 was performed on sagittal sections of lower incisors. lb↔lg indicates the orientation of labial and lingual sides. A, B At E12.5, the Fam20b-deficient dental epithelium started showing more Sox2 expression than normal. C, D At E13.5, the control incisors showed reduced expression of Sox2 in the dental epithelium, while the Fam20b-deficient dental epithelium had strong expression of Sox2 in the lingual side of the enamel organ (arrow). E-H At E14.5 and E15.5, the control incisors gradually lost Sox2 expression from the lingual side of enamel organ. In contrast, the Fam20B-deficient incisors showed strong ectopic Sox2 expression at the lingual side of enamel organ (arrows). Scale bars, 50 μm in A and B, 100 μm in C and D, 200 μm in E and F, 400 μm in G and H. [file 12915_2020_813_MOESM2_ESM.jpg]

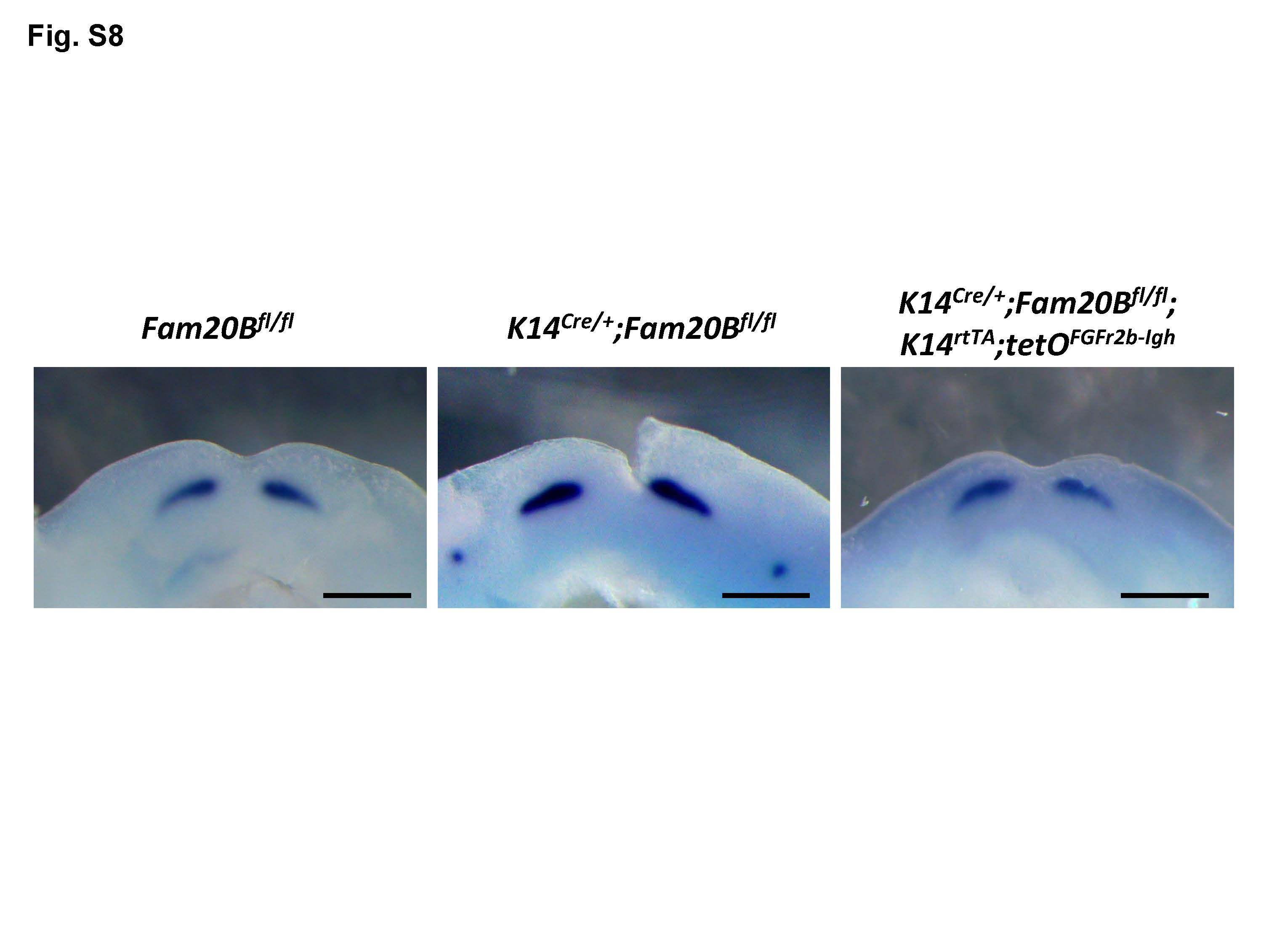

Supplement: Supplementary file 3 — Additional file 3: Figure S3. Inactivation of Sox2 from the dental epithelium partially rescued the supernumerary tooth phenotype in K14Cre/+;Fam20Bfl/fl mice. We introduced Sox2fl/fl allele into K14Cre/+;Fam20Bfl/fl mice to inactivate both Sox2 and Fam20B in the dental epithelium. The double knockout (K14Cre/+;Fam20Bfl/fl;Sox2fl/fl) mice showed reduced number and size of supernumerary teeth (black arrows) compared with Fam20B single-knockout (K14Cre/+;Fam20Bfl/fl; Sox2fl/+) mice. The native teeth lacking Sox2 allele (K14Cre/+;Fam20Bfl/+;Sox2fl/fl) displayed a smaller size than normal (white arrows). [file 12915_2020_813_MOESM3_ESM.jpg]

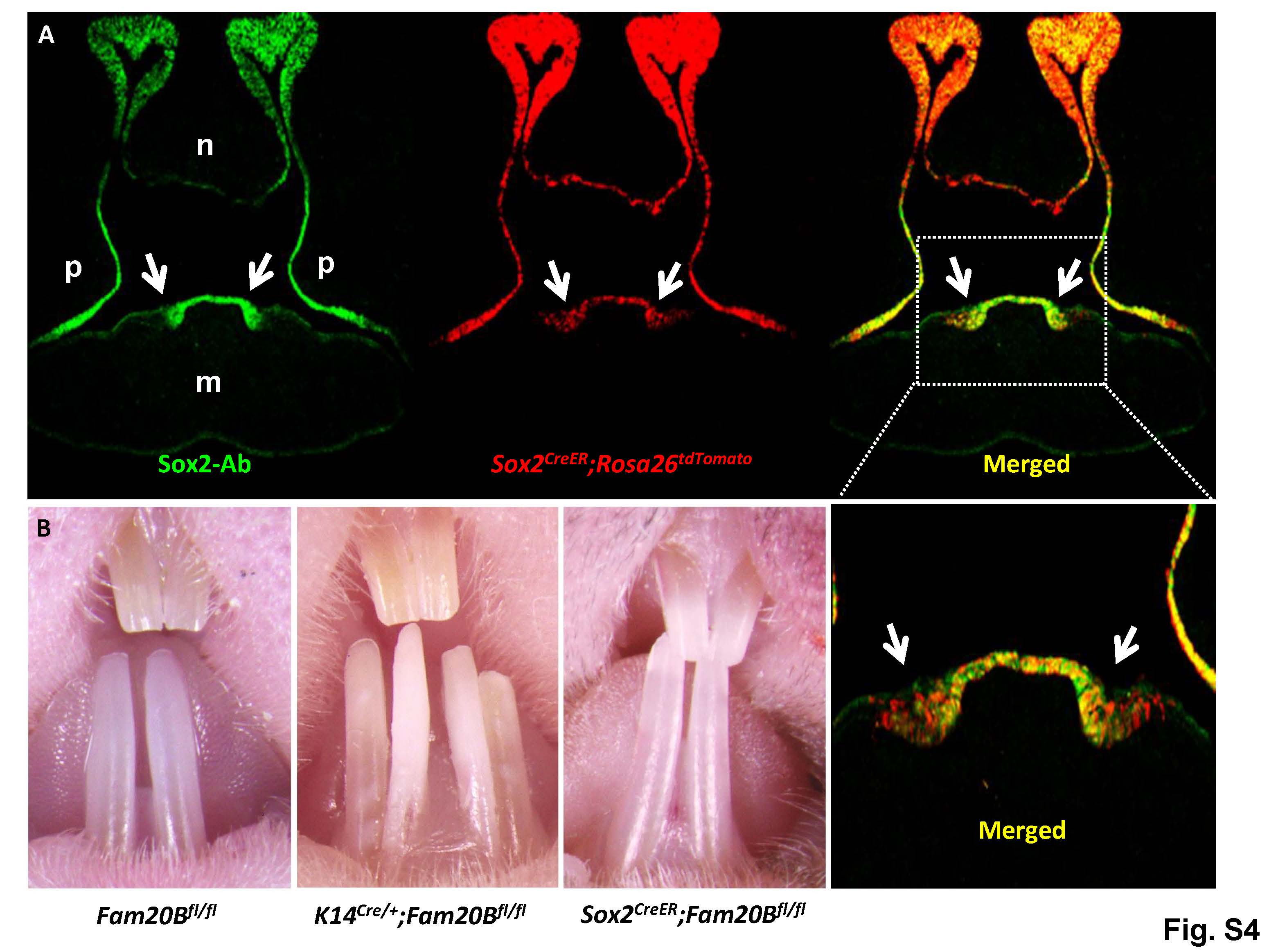

Supplement: Supplementary file 4 — Additional file 4: Figure S4. FAM20B-catalyzed GAGs regulate the homeostasis of Sox2+ cells in a non-autonomous manner. A To validate the efficiency of the Sox2-CreER, we crossbred the CreER line with Rosa26-tdTomato indicator mice and induced the Cre expression with single I.P. injection of tamoxifen at E11.5. The embryos were collected at E12.5 and subjected to cryosection for fluorescence assay. On the same cryosections, Sox2-expressing cells were labeled by immunofluorescence using anti-Sox2 antibody and EGFP-conjugated secondary antibody. The Cre activity indicated by Tomato fluorescence (red) was strongly present in the dental epithelium (arrows) and oral epithelium, as well as nasal mucosa and palatal epithelium. The antibody-labeled Sox2-expressing cells (green) mostly overlapped with CreER active cells (red) and showed yellow on the merged channel. CreER-active cells showed overall broader range than the antibody-labeled Sox2(+) cells in the dental epithelium (especially in the distal side) and nasal mucosa, indicating that the efficiency of Sox2-CreER was strong enough for deleting floxed alleles from the Sox2-expressing cells. n, nose; p, palate; m, mandible. B To determine the regulatory manner of GAGs on Sox2(+) cell homeostasis, we inactivated Fam20B from Sox2(+) lineage using Sox2-CreER. Tamoxifen was administered to mice by i.p. injection at E11.5 and E12.0. Sox2CreER;Fam20Bfl/fl mice did not recapitulate the replacement tooth phenotype, suggesting that GAGs regulate the homeostasis of Sox2(+) cells in a non-autonomous manner. [file 12915_2020_813_MOESM4_ESM.jpg]

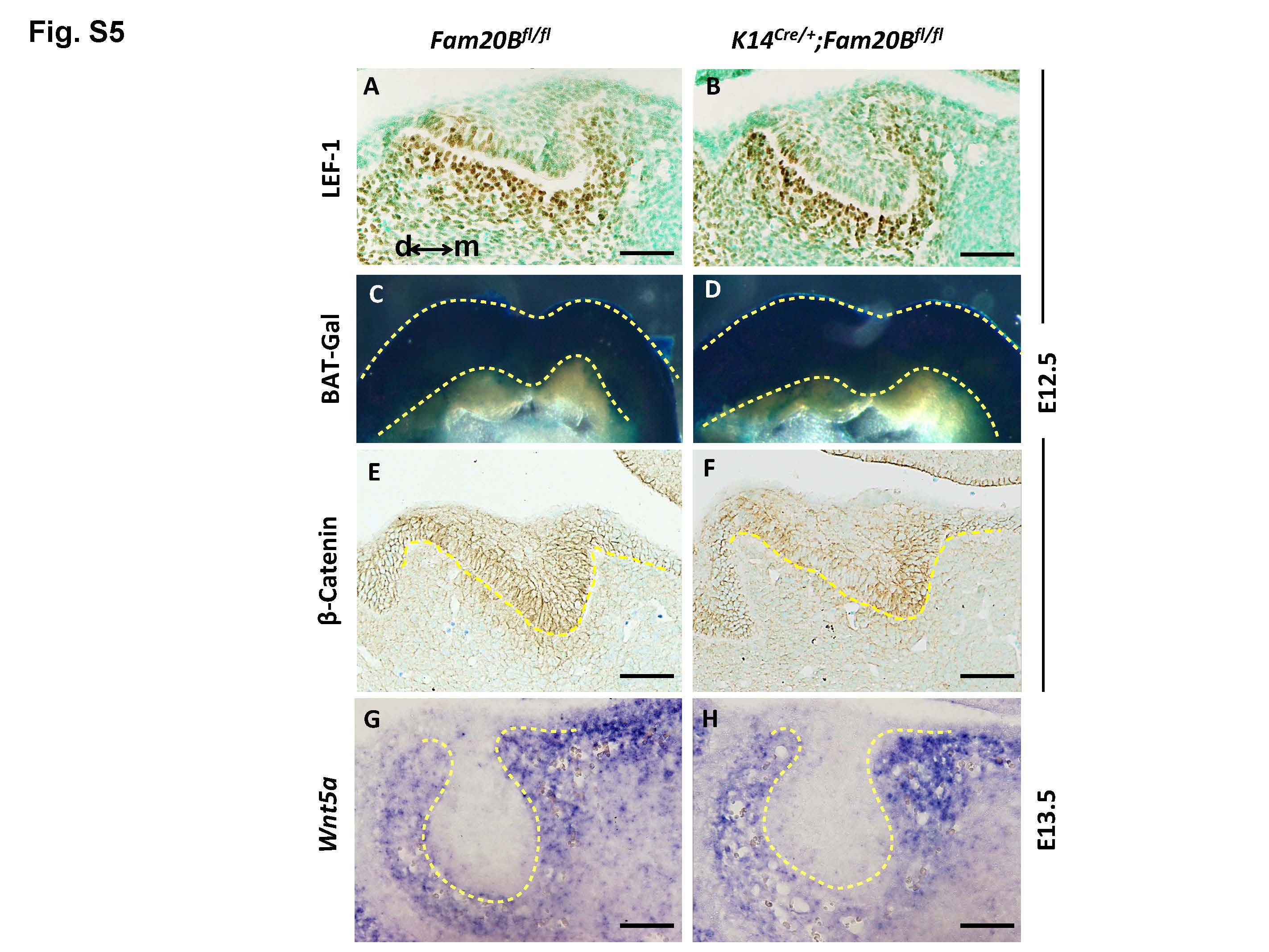

Supplement: Supplementary file 5 — Additional file 5: Figure S5. WNT signaling was not changed in the Fam20B-deficient incisors at the early stage of tooth development. A, B Immunohistochemistry staining of LEF1 on the coronal sections of lower incisors showed no differences between the Fam20B-deficient and control incisors at E12.5. d↔m indicates the orientation of distal and mesial sides. C, D Whole-mount staining of BAT-Gal indicator on E12.5 mandibles showed no differences between the Fam20B-mutants and controls. The yellow dotted lines plotted the areas of LacZ positive staining. E, F Immunohistochemistry staining of β-Catenin on the coronal sections of lower incisors showed no differences between the Fam20B-deficient and control incisors at E12.5. G, H In situ hybridyzation staining of Wnt5a on the coronal sections of lower incisors showed no differences between the Fam20B-deficient and control incisors at E13.5. The yellow dotted lines in E-H indicate the boarder line between the dental epithelium and the dental mesenchyme. Scale bars, 100 μm. [file 12915_2020_813_MOESM5_ESM.jpg]

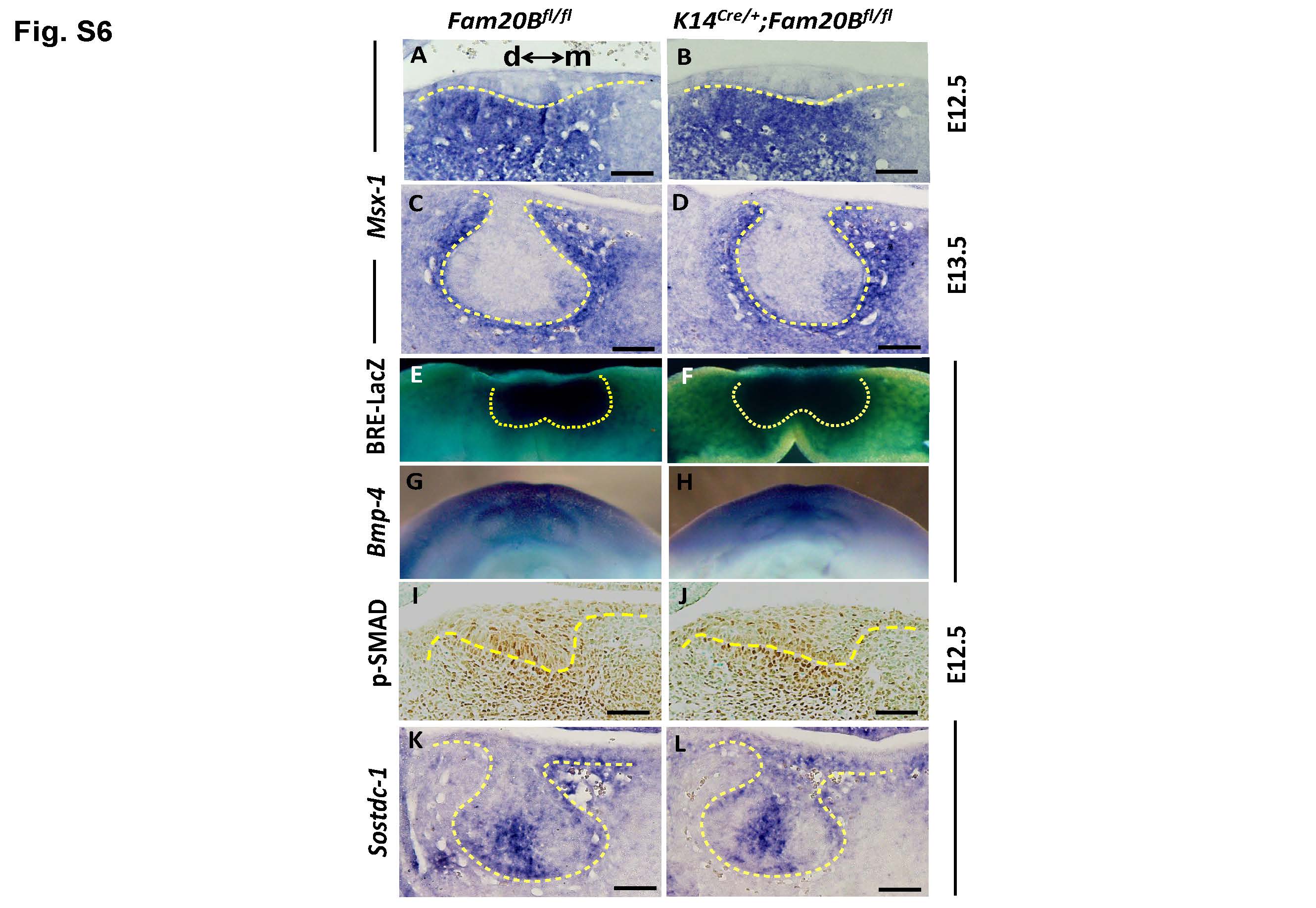

Supplement: Supplementary file 6 — Additional file 6: Figure S6. BMP signaling was not changed in the Fam20B-deficient incisors at the early stage of tooth development. A-D In situ hybridyzation staining of Msx-1 on the coronal sections of lower incisors showed no differences between the Fam20B-deficient and control incisors at E12.5 and E13.5. d↔m indicates the orientation of distal and mesial sides. The yellow dotted lines indicate the boarder line between the dental epithelium and the dental mesenchyme. E, F Whole-mount staining of BRE-LacZ indicator on E12.5 mandibles showed no differences between the incisors of Fam20B-mutants and controls. The yellow dotted lines plotted the areas of LacZ positive staining in the lower incisors. G, H Whole-mount ISH staining of Bmp4 on E12.5 mandibles showed no differences between the incisors of Fam20B-mutants and controls. I, J Immunohistochemistry staining of p-SMAD1/5 on the coronal sections of lower incisors showed no differences between the Fam20B-deficient and control incisors at E12.5. K, L In situ hybridyzation staining of Sostdc-1 on the coronal sections of lower incisors showed no differences between the Fam20B-deficient and control incisors at E12.5. Scale bars, 100 μm. [file 12915_2020_813_MOESM6_ESM.jpg]

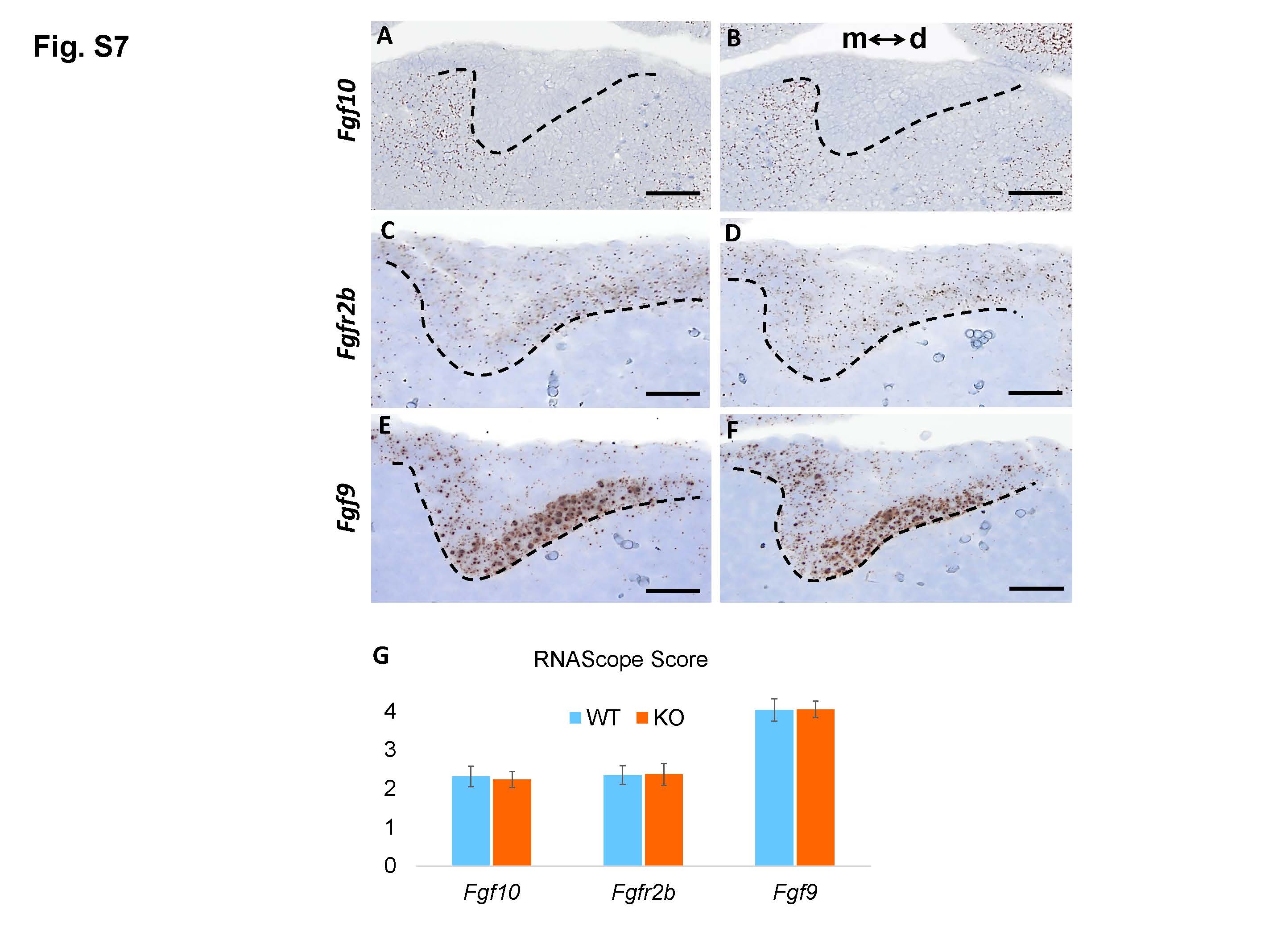

Supplement: Supplementary file 7 — Additional file 7: Figure S7. The transcriptional expression of Fgf10, Fgfr2b and Fgf9 was not changed in the Fam20B-deficient incisors at the early stage of tooth development. A- F RNAScope staining of Fgf10, Fgfr2b and Fgf9 on the coronal sections of lower incisors showed no differences between the Fam20B-deficient and control incisors at E12.5. The dotted lines indicate the boarder line between the dental epithelium and the dental mesenchyme. G Semi-quantitative analysis of RNAScope results showed no significant differences in the transcriptional expression of Fgf10, Fgfr2b and Fgf9 between the Fam20B-deficient (KO) and control (WT) incisors. Scale bars, 100 μm in A and B; 50 μm in C-F. [file 12915_2020_813_MOESM7_ESM.jpg]

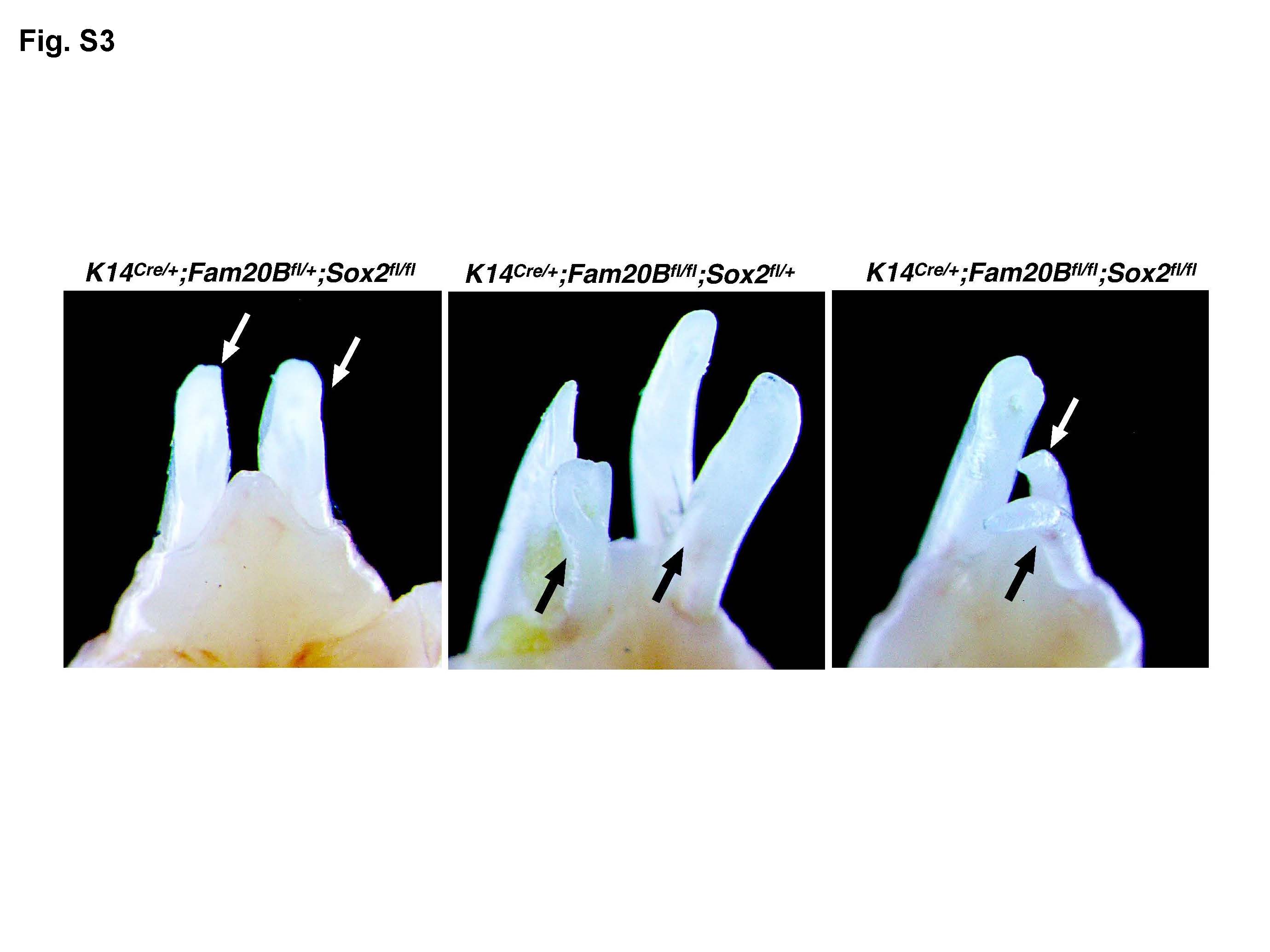

Supplement: Supplementary file 8 — Additional file 8: Figure S8. Inhibition of Fgfr2b in the Fam20B-deficient dental epithelium reduced the expanded expression scope of Shh back to the normal size. Scale bars, 250 μm. [file 12915_2020_813_MOESM8_ESM.jpg]

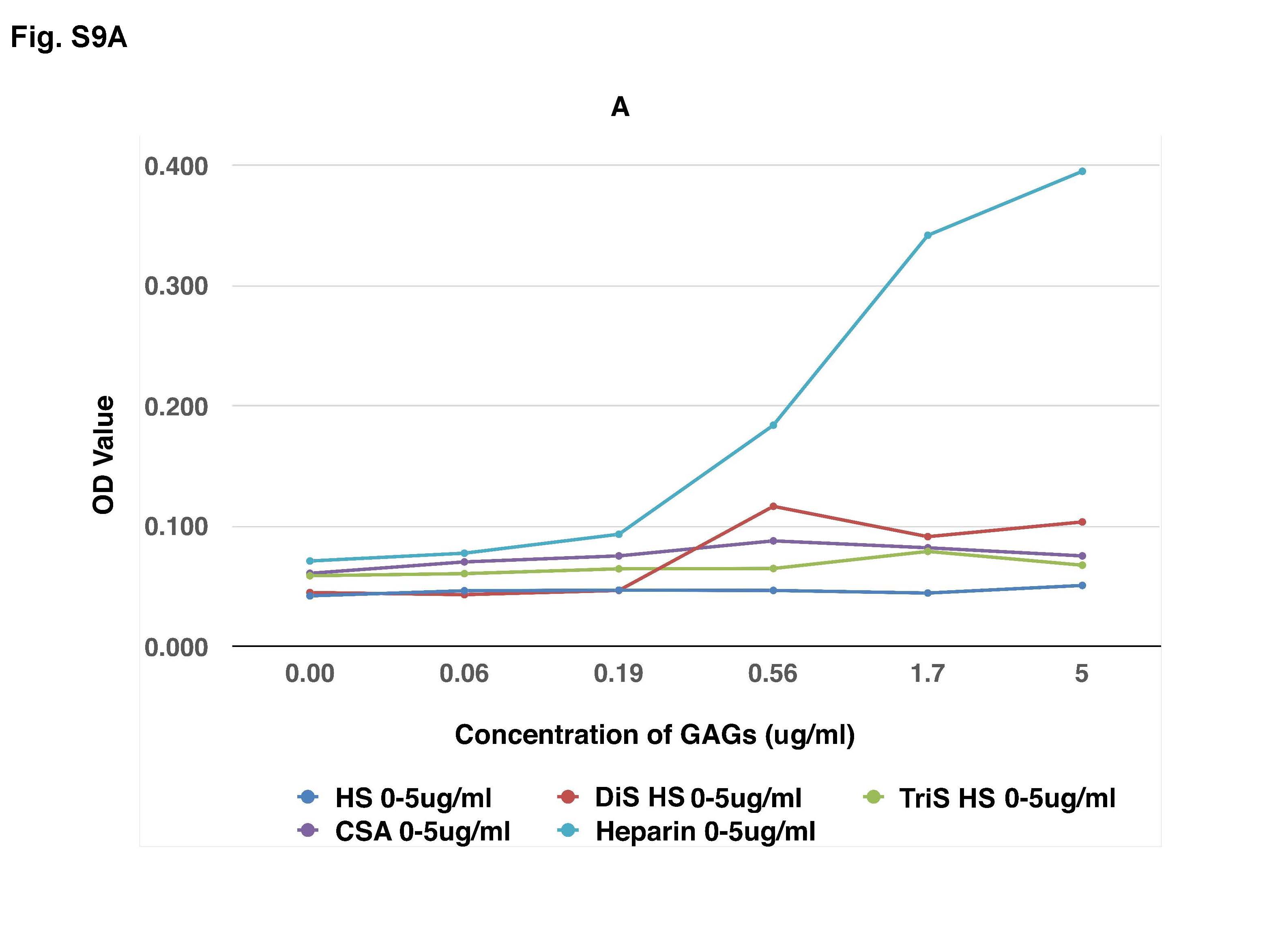

Supplement: Supplementary file 9 — Additional file 9: Figures S9A-S9B. Fig. S9A-[GAGs did not show synergistic effects on FGF10-FGFR2b signaling]. Fig. S9B-[GAGs did not show inhibitory effects on FGF10-FGFR2b signaling]. HS and CS did not show significant synergistic or inhibitory effects on FGF10-FGFR2b signaling in BaF3 cells. A BaF3 cells expressing FGFR2b were cultured in RPMI 1640 media supplemented with 1000 pM FGF10 and 0–5 ng/ml GAGs (HS/HS2S/HS6S/CSA/heparin) for 45 h. Heparin (positive control) showed significant synergistic effects on FGFR2b signaling (P < 0.001), while HS/HS2S/HS6S/CSA groups did not show any significant synergistic effects (P > 0.05). B BaF3-FGFR2b cells were cultured in RPMI 1640 media supplemented with 1000 pM FGF10 and 1.5 μg/ml heparin to boost the baseline of FGFR2b signaling activity (indicated by cell viability OD value). HS/CSA/CSC/CSE/LMW heparin (0–5 ng/ml) supplemented to the culture media did not show any inhibitory effects on FGFR2b signaling (P > 0.05). Two-way ANOVA was used to evaluate the differences among groups. [file 12915_2020_813_MOESM9_ESM.zip › Additional File9_Fig S9a.jpg]

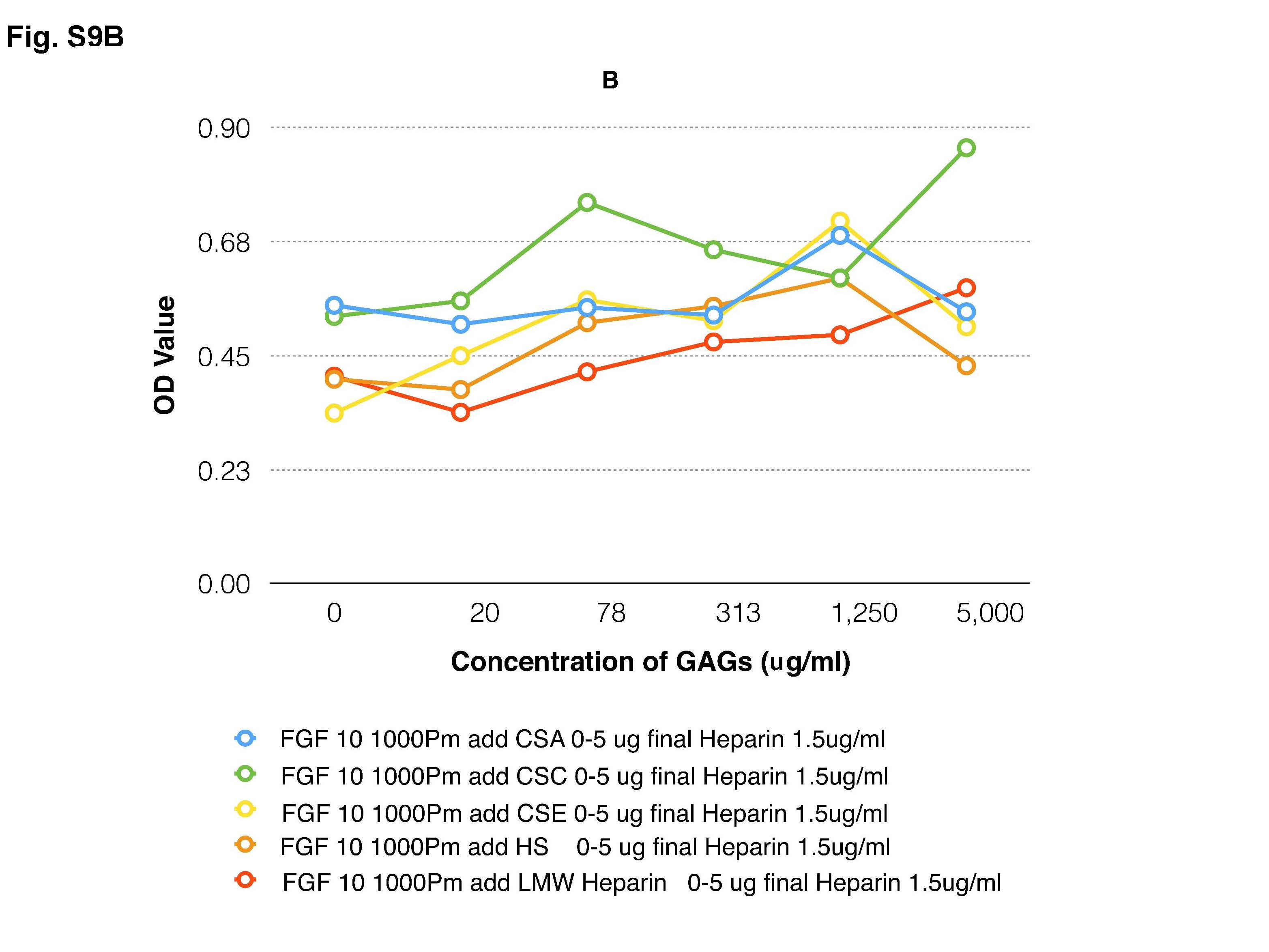

Supplement: Supplementary file 9 — Additional file 9: Figures S9A-S9B. Fig. S9A-[GAGs did not show synergistic effects on FGF10-FGFR2b signaling]. Fig. S9B-[GAGs did not show inhibitory effects on FGF10-FGFR2b signaling]. HS and CS did not show significant synergistic or inhibitory effects on FGF10-FGFR2b signaling in BaF3 cells. A BaF3 cells expressing FGFR2b were cultured in RPMI 1640 media supplemented with 1000 pM FGF10 and 0–5 ng/ml GAGs (HS/HS2S/HS6S/CSA/heparin) for 45 h. Heparin (positive control) showed significant synergistic effects on FGFR2b signaling (P < 0.001), while HS/HS2S/HS6S/CSA groups did not show any significant synergistic effects (P > 0.05). B BaF3-FGFR2b cells were cultured in RPMI 1640 media supplemented with 1000 pM FGF10 and 1.5 μg/ml heparin to boost the baseline of FGFR2b signaling activity (indicated by cell viability OD value). HS/CSA/CSC/CSE/LMW heparin (0–5 ng/ml) supplemented to the culture media did not show any inhibitory effects on FGFR2b signaling (P > 0.05). Two-way ANOVA was used to evaluate the differences among groups. [file 12915_2020_813_MOESM9_ESM.zip › Additional File9_Fig S9b.jpg]
